# Microscopic clusters feature the composition of biochemical tetraspanin-assemblies and constitute building-blocks of tetraspanin enriched domains

**DOI:** 10.1038/s41598-024-52615-1

**Published:** 2024-01-24

**Authors:** Sara C. Schmidt, Annika Massenberg, Yahya Homsi, Dominik Sons, Thorsten Lang

**Affiliations:** Faculty of Mathematics and Natural Sciences, Life & Medical Sciences (LIMES) Institute, University of Bonn, Carl-Troll-Straße 31, 53115 Bonn, Germany

**Keywords:** Biophysics, Cell biology

## Abstract

Biochemical approaches revealed that tetraspanins are multi-regulatory proteins forming a web, where they act in tetraspanin-enriched-microdomains (TEMs). A microscopic criterion differentiating between web and TEMs is lacking. Using super-resolution microcopy, we identify co-assemblies between the tetraspanins CD9 and CD81 and CD151 and CD81. CD9 assemblies contain as well the CD9/CD81-interaction partner EWI-2. Moreover, CD9 clusters are proximal to clusters of the CD81-interaction partner CD44 and CD81-/EWI-2-interacting ezrin–radixin–moesin proteins. Assemblies scatter unorganized across the cell membrane; yet, upon EWI-2 elevation, they agglomerate into densely packed arranged-crowds in a process independent from actin dynamics. In conclusion, microscopic clusters are equivalent to biochemical tetraspanin-assemblies, defining in their entirety the tetraspanin web. Cluster-agglomeration enriches tetraspanins, which makes agglomerations to a microscopic complement of TEMs. The microscopic classification of tetraspanin assemblies advances our understanding of this enigmatic protein family, whose members play roles in a plethora of cellular functions, diseases, and pathogen infections.

## Introduction

Tetraspanins are small membrane proteins that played an evolutionary role in the unicell-to-multicell transition^1^. Widely studied tetraspanins are known as ‘cluster of differentiation’ (CD) molecules CD9, CD37, CD53, CD63, CD81, CD82, and CD151, employing a nomenclature not referring to function, but a cell-surface-molecule immunophenotyping-protocol. The human genome includes 33 family members^2,3^, from which only a minority is studied in depth. Yet, we face a broad range of roles. Those include trafficking, signaling, regulation of protease activity, cell proliferation, adhesion, spreading, migration, cell–cell fusion, and extracellular vesicle formation^4–9^. Additional pathophysiological roles are documented in cancer, neurodegeneration and infectious diseases^3,10,11^. The large functional diversity grounds on the capacity of tetraspanins to interact with different types of partner proteins, like immunoglobulin superfamily proteins, proteases, integrins, and several other receptor types. Tetraspanins also interact among each other.

Dissection of the tetraspanin-interaction-network began three decades ago, employing immunoprecipitation after cell lysis with detergents of different strengths^12^. Overexpression of one tetraspanin influences the tetraspanin ratio in co-precipitates, which lead to the important conclusion that tetraspanins compete between each other in multimeric-tetraspanin-assemblies^13^. Such multimers explain why antibodies against different tetraspanins trigger the same cellular response. For example, CD9-, CD53-, CD81- or CD82-antibodies all stimulate T-cell activation^14^, and antibodies to CD9, CD81, and CD82 all induce homotypic aggregation^13^. These, and many more similar observations, document that tetraspanins and their partners form a network of interactions, originally referred to as the ‘tetraspan network’^13^, and later as the ‘tetraspanin web’. A decade later, it was proposed that tetraspanins organize in signaling platforms that were named TEMs^15^. The acronym TEMs, as it stands for ‘tetraspanin enriched microdomains’, implies that tetraspanins locally enrich in order to exert a special function.

The TEM model links tetraspanin-function to tetraspanin-location in a specialized plasmalemmal domain. However, why tetraspanins function via TEMs remains largely unclear. In TEMs, tetraspanins may stabilize their interaction partners, assist in their folding, direct them to their cellular destination, or regulate their activity. Moreover, TEMs may constitute scaffolds for membrane curvature in trafficking^16,17^. On the quest to understand TEMs, the finding that one family member can produce opposite effects causes substantial confusion. For instance, CD9 typically promotes tumor suppression but in certain cell types it has an oncogenic function^15^. Most likely, differently composed TEMs do form in different cellular environments.

For understanding the mechanisms of TEM-function, we need a more detailed picture about their nano-scale organization. In accordance with biochemistry, in a single-particle-tracking study, CD9 molecules transiently locate to interaction platforms enriched in CD9 and its binding partners^18^, suggesting that an equilibrium underlies the formation of tetraspanin assemblies. A palmitoylation deficient CD9 mutant is less confined to tetraspanin enriched areas^18^, again in line with biochemistry, showing that palmitoylation stabilizes tetraspanin assemblies^19–21^.

For studying tetraspanin-organization, also dSTORM was applied. In this super-resolution-microscopy method, an image is the sum of lots of single-molecule localizations, in which superlocalization determines molecule positions with ⁓ 10 nm precision. An algorithm assigns single-molecules to clusters of varying shape and size. With this methodology, it was shown that overexpression of CD82 only moderately increases the size of CD82 clusters, but the cluster size of a palmitoylation-deficient mutant triples^22^. Hence, like single-particle-tracking, also dSTORM suggests that palmitoylation is required for tight clustering. Moreover, palmitoylation is required for the tight packing of α4 integrin subunits into clusters^23^. Similarly, CD82 controls nanoscale organization of N-cadherin^22^. Altogether, dSTORM provides interesting details that provide explanations for biochemical observations.

STED microscopy is a super-resolution microscopy technique that, different from STORM, it is not capable of resolving single molecules in a dense crowd. Instead, a cluster of proteins, that in dSTORM is an algorithm-computed group of molecules, is imaged as a single spot. This is a disadvantage; on the other hand, STED microscopy is independent of image processing algorithms. In STED microscopy, tetraspanin-clusters of CD53 and CD37 showed only minor overlap with CD81-/CD82-clusters, suggesting segregation of different tetraspanins and their interaction partners into different plasmalemmal entities^24^. Hence, clusters apparently are defined mainly by a single molecular component, what distinguishes them from biochemically characterized tetraspanin-multimers.

Here, we set out for clarifying the relationship between microscopic clusters and biochemically characterized tetraspanin-assemblies. As example, we examine the ubiquitously expressed and widely studied sister tetraspanins CD9 and CD81, and the tetraspanin CD151. Regarding tetraspanin interaction partners, we study the CD9 and CD81 primary interaction partner EWI-2^25^, the CD81 interaction partner CD44^26^, and the CD81/EWI-2/EWI-F interaction partner ERM^27^.

## Results

### Co-assembly of tetraspanins

**Figure 1:**
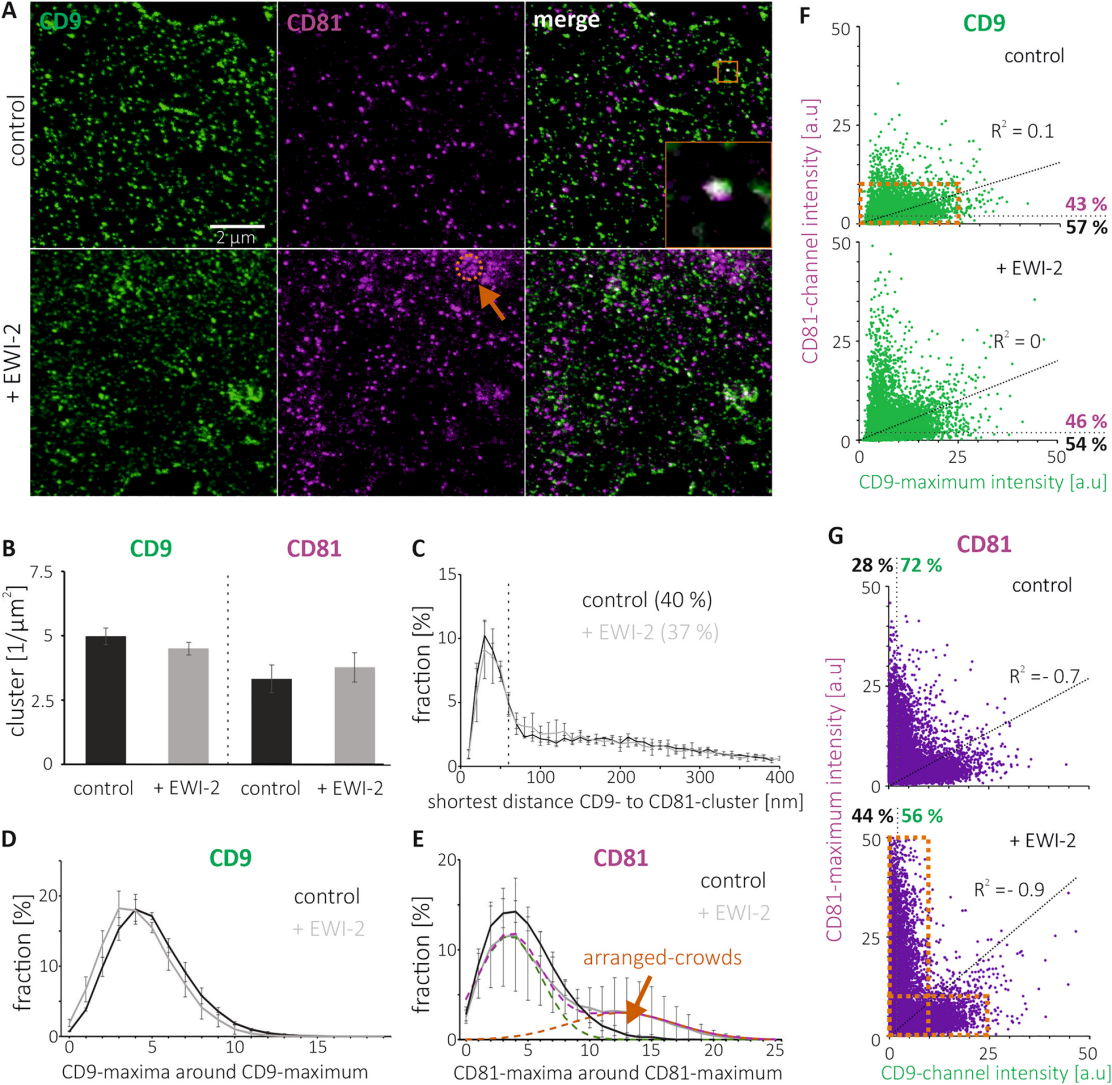
Cluster density, shortest inter-cluster distance, number of neighbored-maxima and maxima inter-channel intensity correlation of CD9 and CD81 in control cells and after EWI-2 elevation. (**A**) STED micrographs of membrane sheets from HaCaT cells (‘control’) or EWI-2-GFP overexpressing HaCaT cells (‘+ EWI-2’). Membranes are fixed and stained for CD9 (Alexa594; shown in green) and CD81 (STAR RED; shown in magenta). During image acquisition, membranes from control and EWI-2-GFP overexpressing cells were identified via F-actin staining (with Phalloidin-iFluor 488) and the GFP-tag (GFP-fluorescence + Atto488-labelled GFP-nanobody), respectively. Prior to STED imaging, from all channels confocal micrographs were taken (for clarity we show example images from the green channel only in Fig. 4A). Orange arrow points to a 900 nm circle placed at a location with crowded maxima. Orange box in the upper right marks magnified view. (**B**) CD9- and CD81-clusters density. (**C**) Percentage of CD9-clusters plotted against distance to the next nearest CD81-cluster. The dashed vertical line marks 60 nm. Clusters with distances ≤ 60 are counted as overlapping clusters; the fractions are stated as percentages. (**D**–**E**) Percentage of (**D**) CD9- and (**E**) CD81-maxima plotted against the number of their neighbored-maxima located in a 900 nm diameter circle. (**E**) The dashed purple line retraces the ‘+ EWI-2’ distribution and is the sum of two Gaussian distributions: green (68% of the area below the dashed purple line) and orange (32%). Values are given as means ± SD (n = 3 biological replicates). (**F**–**G**) The intensity of a (**F**) CD9- and (**G**) CD81-maximum, measured in a 100 nm diameter circular ROI, plotted against the intensity in the respective other channel. For clarity, maxima intensities ≥ 50 a.u. are not shown. Top, control; bottom, after EWI-2 elevation. R^2^ values refer to linear regression lines (see diagonal dotted lines) fitted to the data points and through the origin. Dotted lines parallel to the axes separate maxima with intensities below the noise level (≤ 2 a.u.). Percentage values refer to maxima above (magenta/green) and below (black) the noise level. For magnified views, see Supplementary Fig. 5. Please note that EWI-2 increases in this experiment the CD81-maxima intensity by 79% (compare also upper and lower panels of (**G**)), wherefore in (**A**), at the same scaling, CD81 spots are better recognized in the image of EWI-2 overexpressing cells.

For studying the nano-scale organization of tetraspanin assemblies, we employ superresolution STED microscopy on immunostained HaCaT cell membranes from control and EWI-2 overexpressing cells. We use membrane sheets to avoid long detergent-treatments that may change the distribution and packing of membrane proteins. In fluorescence micrographs of CD9-/CD81-stained membranes, we find CD9-/CD81-signals concentrated in spots of variable brightness, scattered across the cell membrane (Fig. 1A). Compared to conventional resolution, in STED micrographs spots are sharper and better resolved (Supplementary Fig. 1). In the overlays of STED micrographs, some ‘green’ CD9- and ‘magenta’ CD81-spots form ‘white’ spots (for an example see magnified view in Fig. 1A). This suggests concentric overlap, or in other words, that CD9 and CD81 locate together in the same assembly.

For analysis, an image-analysis-algorithm detects local maxima yielding maxima positions in pixel coordinates (Supplementary Fig. 2A). Using a 100 nm diameter circular ROI (Supplementary Fig. 2C), the intensity at the maximum position is measured and background corrected by the average intensity measured next to the membrane sheet. The same circular ROI measures the fluorescence mass centers, resulting in more precise subpixel coordinate positions of the maxima. In addition, we place a 31-pixel long and 3-pixel width linescan horizontally and vertically onto every detected local maxima to measure its intensity profile (Supplementary Fig. 2B and E). The best Gaussian fit is selected, and its full width at half maximum (FWHM) defines the maximum size. Maxima with a fit quality of R^2^ > 0.8 and the Gaussian peaks positioned within the central 10 pixels of the 31-pixel intensity profile are considered as clearly defined maxima, to which we refer in the text as ‘clusters’. In the distance analysis (see e.g. Fig. 1C), only maxima rated as clusters are included, using the subpixel coordinate position (mass center). All maxima are included in the inter-channel intensity correlations (see e.g. Fig. 1F and G) and the neighbored-maxima distributions (see e.g. Fig. 1D and E).

To characterize the maxima, we plot from the maxima of Fig. 1 the intensity and FWHM versus the R^2^ value. EWI-2 elevation has no effect on CD9-/CD81-maxima density and CD9-maxima intensity, but increases the CD81-maxima intensity by 79% (Supplementary Fig. 3). More than half of the CD9- and CD81-maxima have an R^2^ > 0.8. These maxima are between 37% and 174% brighter than maxima with an R^2^ ≤ 0.8. Please note that maxima with an R^2^ > 0.8 are not equal to clusters, as for cluster classification additionally the centered peak criteria applies (see above). However, the second criterium has only a small influence which is why most maxima with an R^2^ > 0.8 rate as clusters. Within the population of maxima with an R^2^ > 0.8, we observe a positive trend between intensity and R^2^ value. We assume this is because brighter maxima are associated with a higher signal-to-noise ratio, and therefore their intensity profile fits better to a Gaussian distribution. In contrast, we observe no correlation between the size (FWHM) of maxima, which is in the range of 100 nm, and R^2^ value (Supplementary Fig. 3). However, the resolution limit of our STED microscope ranges between 65 and 100 nm^28^. As a result, we largely overestimate the size and may not resolve, if there is any, relationship between size and R^2^. On the biological level, clusters, that tend to be brighter maxima, possibly contain more molecules, in contrast to dim maxima, that may represent low oligomers/single molecules. Alternatively, instead of harboring more molecules, they may contain tetraspanins in a conformation better recognized by the antibody. In particular, this could apply for the CD81 antibody that recognizes after EWI-2 elevation not more but clearly brighter maxima. To exclude any major unspecific staining, we tested for background staining of the polyclonal CD81 antibody by staining CD81-lacking HepG2 cell membranes. We find 1/40 of the maxima per µm^2^ when compared to HaCaT cells (Supplementary Fig. 4). These maxima tend to be very bright and essentially all of them rate as clusters, which is why in the following we overestimate the CD81-cluster density by 0.28 clusters per µm^2^.

In control and EWI-2 overexpressing cells, we find 3 to 5 CD9-/CD81-clusters per µm^2^ (Fig. 1B). Mixed clusters of CD9 and CD81 should have concentric CD9-/CD81-signals (concentric = the distance between their positions is zero). However, the distribution of shortest distances between CD9- and CD81-clusters does not peak at zero but at 30 nm (Fig. 1C), which likely is due to the resolution limit (see above). Hence, even if signals arise from the same location, we cannot expect them to be perfectly concentric. The distance distribution from 0 to 100 nm (Fig. 1C) features a normal distribution with a peak at 30 nm and a basis of 60 nm. Based on the above-mentioned technical limitations, we assume the peak at 30 nm represents the population of CD9 that co-assembles with CD81. It accounts for 40% and 37% in control cells and EWI-2-overexpressing cells, respectively (Fig. 1C).

Provided assemblies are thermodynamically controlled CD9/CD81-mixtures, the CD9 and CD81 copy number per assembly may correlate. Using CD9-/CD81-intensities as indirect and relative read-out of copy number, we analyse in the following the relationship between the CD9- and CD81-intensities at CD9- or CD81-maxima positions. In Fig. 1F, at the location of a CD9-maximum we measure the CD9-intensity in a circular ROI of 100 nm diameter. Using the same ROI, in the CD81-channel we measure the CD81-intensity, and plot the intensities against each other. In control and overexpressing cells, fitting of a linear regression line through the origin yields R^2^ values of 0.1 and 0 (no significant correlation). Visually, data points rather distribute parallel to the abscissa. In general, CD9-intensities do not exceed 25 a.u. and CD81-intensities are in the range below 10 a.u. (see orange box in Fig. 1F, top). Considering an intensity of ≤ 2 a.u as noise level, in the control, 57% of the data points are CD9-only maxima (see supplementary Fig. 5 for a magnified view of Fig. 1F). Conversely, 43% contain both proteins, a value in accordance with the 40% overlap from the shortest distance analysis (Fig. 1C). EWI-2 has no effect on the size of these fractions (46% of maxima containing both proteins (Fig. 1F); 37% of overlapping clusters (Fig. 1C)). The distribution of CD9 data points may be consistent with only one population of CD9-maxima. However, more than half of the maxima are CD9-only maxima, pointing towards the possibility that CD9-only-maxima may constitute a separate population.

In Fig. 1G, we measure at the location of a CD81-maximum the CD81-intensity in a circular ROI and plot it against the respective intensity in the CD9-channel. Linear regression line fitting yields negative R^2^ values of − 0.7 and − 0.9, indicating segregation of data points into distinct populations, which also can be recognized visually in the data points distribution (see the two orange boxes in Fig. 1G, bottom; for a magnified presentation of data points see Supplementary Fig. 5). One of the CD81-maxima populations has the CD81- and CD9-intensity up to 10 and 25 a.u., respectively, like the above-mentioned CD9-maxima population. The second CD81-maxima population is stronger in CD81-intensity (sometimes clearly exceeding 25 a.u., in particular after EWI-2 overexpression) but remains below 10 a.u. in the CD9-channel (upright orange box in Fig. 1G). In conclusion, we can differentiate between two CD81-maxima populations, one containing relatively more CD81 (up to 50 a.u.) and less CD9 (up to 10 a.u.), and one with less CD81 (up to 10 a.u.) and more CD9 (up to 25 a.u.). The fractions of CD81-maxima positive for CD9 correspond to 72% (control) and 56% (+ EWI-2; Fig. 1G). Thus, we propose there are at least two types of CD81-assemblies differing in their CD81:CD9 ratio.

**Figure 2:**
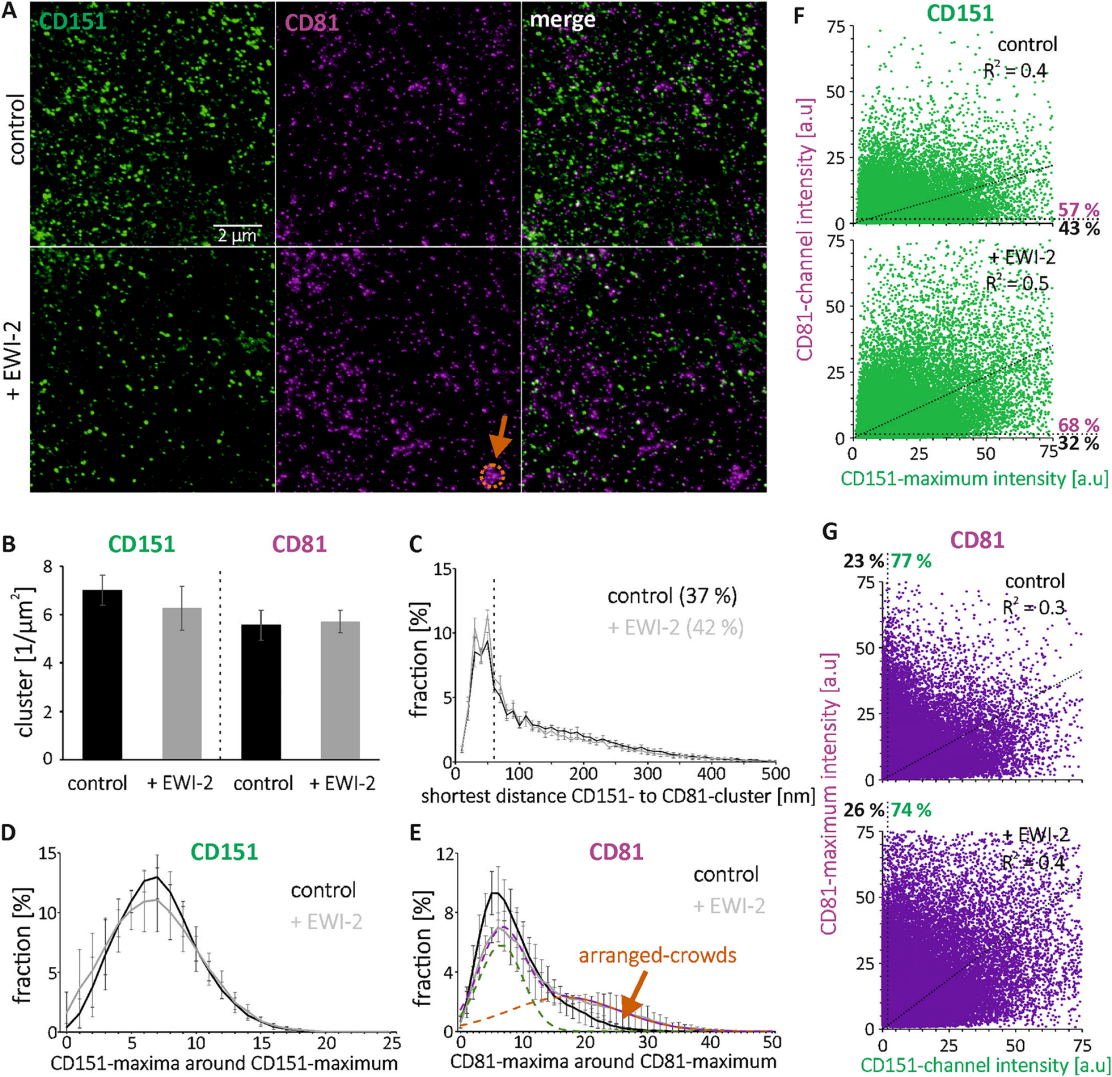
Cluster density, shortest inter-cluster distance, number of neighbored-maxima and maxima inter-channel intensity correlation of CD151 and CD81 in control cells and after EWI-2 elevation. (**A**) As in Fig. 1, but analysis of CD151 (Alexa594; shown in green) and CD81 (STAR RED; shown in magenta). Shown are STED micrographs of membrane sheets from HaCaT cells (‘control’) or EWI-2-GFP overexpressing HaCaT cells (‘+ EWI-2’). Orange arrow points to a 900 nm circle placed at a location with crowded maxima. (**B**) CD151- and CD81-clusters density. (**C**) Percentage of CD151-clusters plotted against distance to the next nearest CD81-cluster. The dashed vertical line marks 60 nm. Clusters with distances ≤ 60 are counted as overlapping clusters; the fractions are stated as percentages. (**D**–**E**) Percentage of (**D**) CD151- and (**E**) CD81-maxima plotted against the number of their neighbored-maxima located in a 900 nm diameter circle. (**E**) The dashed purple line retraces the ‘+ EWI-2’ distribution and is the sum of two Gaussian distributions: green (50% of the area below the dashed purple line) and orange (50%). Values are given as means ± SD (n = 3 biological replicates). (**F**–**G**) The intensity of a (**F**) CD151- and (**G**) CD81-maximum, measured in a 100 nm diameter circular ROI, plotted against the intensity in the respective other channel. For clarity, maxima intensities ≥ 75 a.u. are not shown. Top, control; bottom, after EWI-2 elevation. R^2^ values refer to linear regression lines (see diagonal dotted lines) fitted to the data points and through the origin. Dotted lines parallel to the axes separate maxima with intensities below the noise level (≤ 2 a.u.). Percentage values refer to maxima above (magenta/green) and below (black) the noise level. Please note that EWI-2 increases the CD81-maxima intensity (compare upper and lower panel of (**G**)), wherefore in (**A**) CD81 spots are better recognized in the image of EWI-2 overexpressing cells.

CD9 and CD81 exhibit a high degree of sequence homology^16^ and share EWI-2 as a primary interaction partner^25^. Therefore, they may behave similarly, explaining why in control cells 40% of the CD9 clusters overlap with CD81 clusters (Fig. 1C) and more than 40% of the CD9 maxima are CD81 positive (Fig. 1F). We next wondered whether a distinct tetraspanin, as CD151, co-assembles with CD81 as well. In the shortest distance analysis, we find again a sharp peak. The overlap of CD151- with CD81-clusters is 37% in the control and 42% in EWI-2 overexpressing cells (Fig. 2C). Altogether, the data suggest CD151 co-assembles with CD81 equally well as CD9 and CD81.

### Co-assembly of CD9 with TEM components

**Figure 3:**
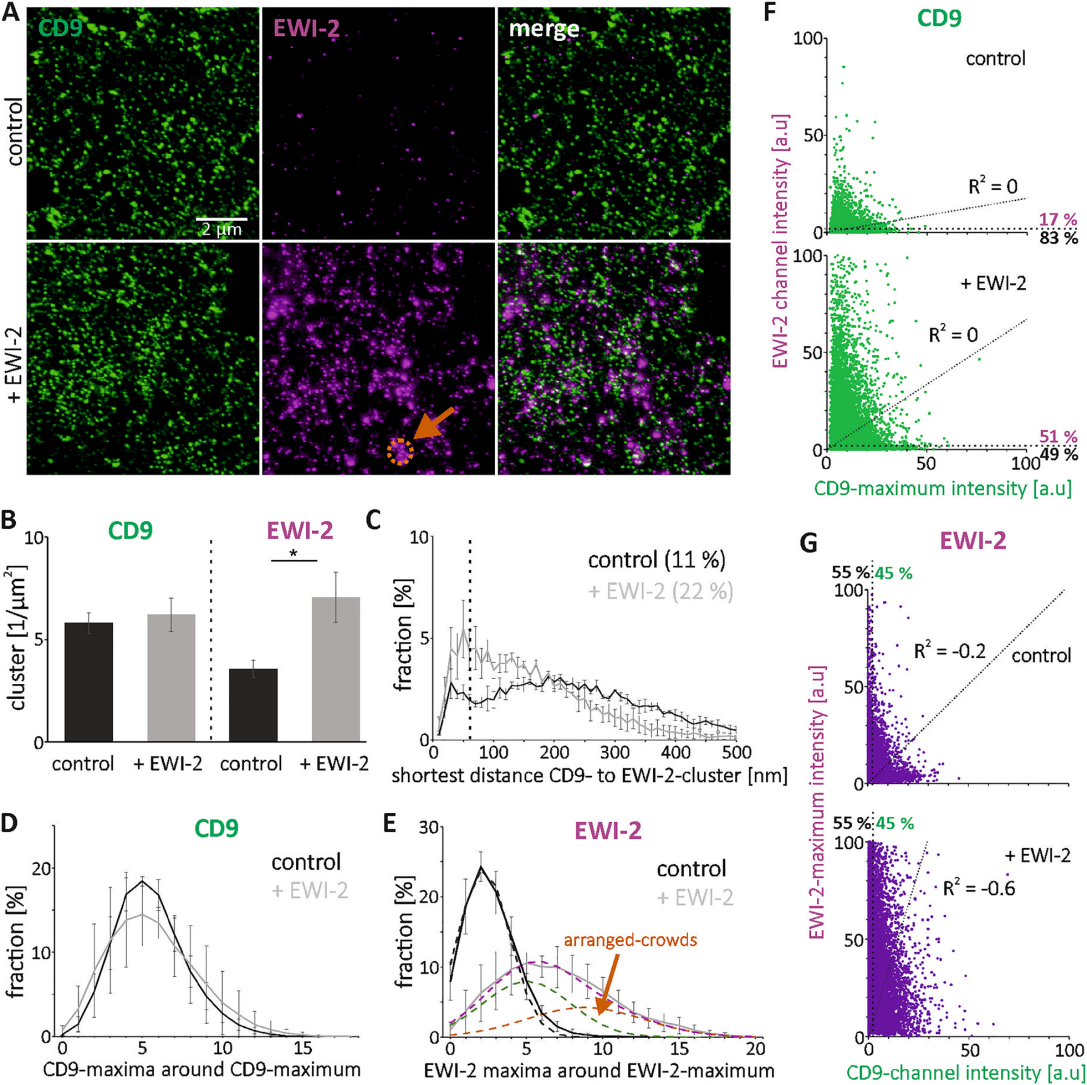
Cluster density, shortest inter-cluster distance, number of neighbored-maxima and maxima inter-channel intensity correlation of CD9 and EWI-2 in control cells and after EWI-2 elevation. (**A**) As in Fig. 1, but analysis of CD9 (Alexa594; shown in green) and EWI-2 (STAR RED; shown in magenta). Shown are STED micrographs of membrane sheets from HaCaT cells (‘control’) or EWI-2-GFP overexpressing HaCaT cells (‘+ EWI-2’). Orange arrow points to a 900 nm circle placed at a location with crowded maxima. (**B**) CD9- and EWI-2-clusters density. (**C**) Percentage of CD9-clusters plotted against distance to the next nearest EWI-2-cluster. The dashed vertical line marks 60 nm. Clusters with distances ≤ 60 are counted as overlapping clusters; the fractions are stated as percentages. (**D**–**E**) Percentage of (**D**) CD9- and (**E**) EWI-2-maxima plotted against the number of their neighbored-maxima located in a 900 nm diameter circle. (**E)** Dashed black line: Gaussian distribution fitted to control distribution (continuous black line). The dashed purple line retraces the ‘+ EWI-2’ distribution and is the sum of two Gaussian distributions: green (58% of the area below the dashed purple line) and orange (42%). Values are given as means ± SD (n = 3 biological replicates). (**F**–**G**) The intensity of a (F) CD9- and (G) EWI-2-maximum, measured in a 100 nm diameter circular ROI, plotted against the intensity in the respective other channel. For clarity, maxima intensities ≥ 100 a.u. are not shown. Top, control; bottom, after EWI-2 elevation. R^2^ values refer to linear regression lines (see diagonal dotted lines) fitted to the data points and through the origin. Dotted lines parallel to the axes separate maxima with intensities below the noise level (≤ 2 a.u.). Percentage values refer to maxima above (magenta/green) and below (black) the noise level.

Next, we examined whether CD9-clusters also overlap with the CD9 interaction partner EWI-2, and if so, whether the degree of overlap depends on the EWI-2 concentration. As in Fig. 1B, EWI-2-overexpression does not change the CD9-cluster density, but doubles the EWI-2-cluster-density (Fig. 3B). Hence, clusters of endogenous EWI-2 do not adsorb overexpressed EWI-2 (e.g. by forming larger clusters), but instead novel clusters do form. The fraction of CD9-clusters overlapping with EWI-2-clusters is 11% and doubles after EWI-2 overexpression (22%; Fig. 3C). The maxima inter-channel intensity correlations are not significant (R^2^ values of 0 to – 0.6; Fig. 3F and G). Moreover, there is no indication that data points may split up into two populations.

In control cells, 17% of the CD9-maxima are EWI-2 positive (Fig. 3F). EWI-2 elevation more than doubles this fraction to 51%, in line with the shortest distance analysis, where we see a doubling in overlap (from 11 to 22%; Fig. 3C). From the perspective of EWI-2-maxima, about half of the maxima contain CD9, independent from EWI-2-elevation (Fig. 3G). Yet, as in this experiment EWI-2 elevation increases the number of maxima, it also increases the absolute number of CD9-positive EWI-2 maxima from 4135 to 7562. In conclusion, CD9 and its interaction partner EWI-2 do co-assemble, although not to the same extent as CD9 and CD81 or CD151 and CD81 do. Moreover, an increase of EWI-2 promotes the overlap between CD9 and EWI-2, albeit it should be pointed out that likely this is not due to an altered binding affinity, but simply due to more EWI-2 that can interact with CD9.

CD44 is also a ‘cluster of differentiation’ molecule, but not a tetraspanin. It is a cell surface glycoprotein involved in cell–cell interactions, cell adhesion and migration, processes, in which tetraspanins play a role. Recently, protein structure modeling and interface prediction-guided mutagenesis demonstrated that CD81 is an interaction partner of CD44^26^. Hence, different from EWI-2, CD44 binds only to CD81. In the following, we ask whether CD9-assemblies overlap also with CD44.

Analyzing the shortest distance of CD9-clusters to CD44-clusters, there is no peak at 30 nm, but a broad maximum between 50 and 70 nm (Supplementary Fig. 6C). This distance range is close to cluster sizes reported by others using the same microscopy technique^24^. Hence, CD9- and CD44-clusters do not concentrically overlap, but the data suggests they are as proximal as their physical size allows, implying segregation of CD9 and CD44 into different membrane entities that, however, are closely associated.

**Figure 4:**
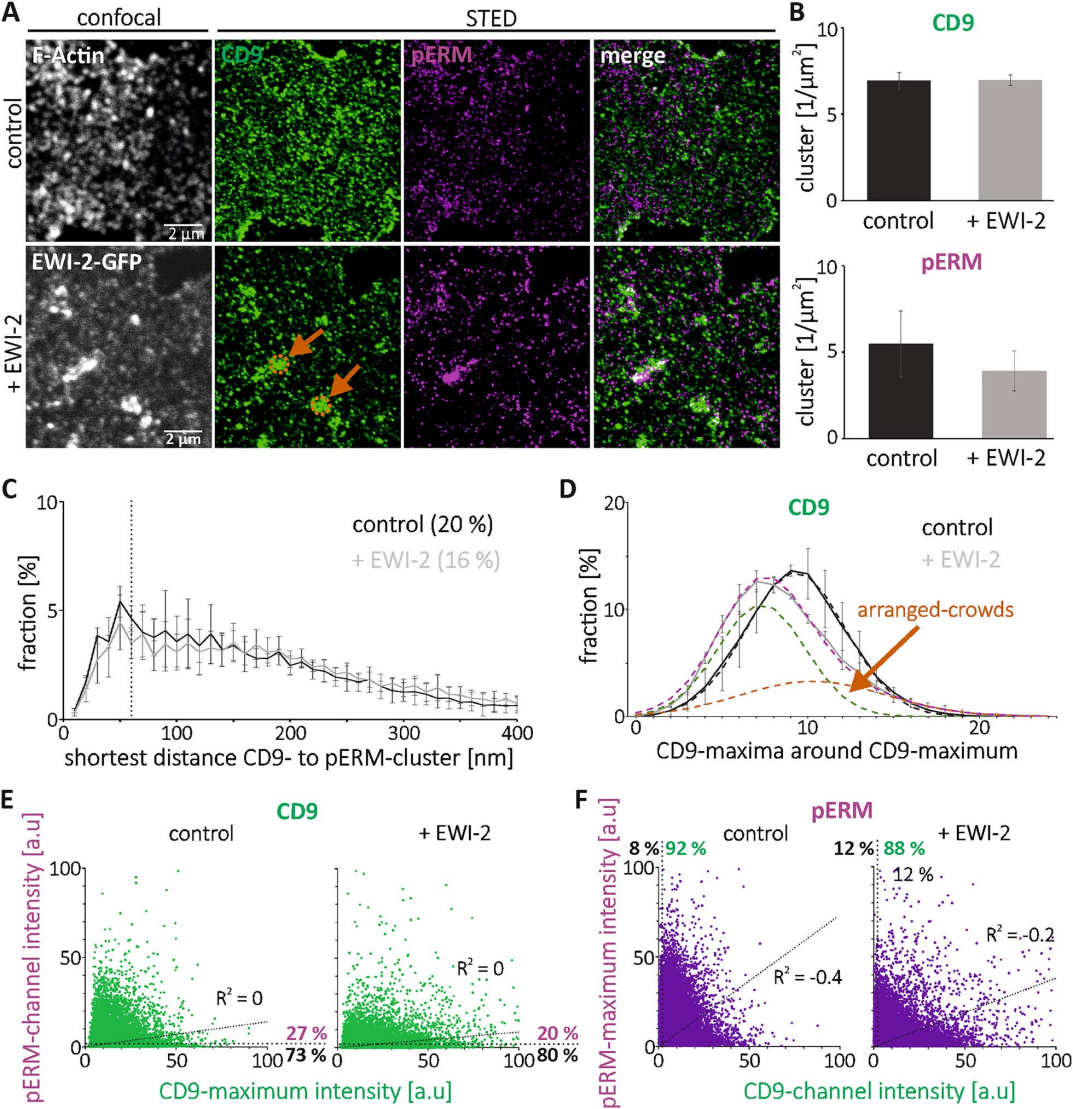
Cluster density, shortest inter-cluster distance, number of neighbored-maxima and maxima inter-channel intensity correlation of CD9 and pERM in control cells and after EWI-2 elevation. (**A**) As in Fig. 1, but analysis of CD9 (Alexa594; shown in green) and pERM (STAR RED; shown in magenta). From HaCaT cells (‘control’) and EWI-2-GFP overexpressing HaCaT cells (‘ + EWI-2’), confocal micrographs of actin staining (‘control’) and nanobody enhanced GFP-signal (‘+ EWI-2’), and STED micrographs of CD9 and pERM are shown. The occurrence of bright EWI-2-GFP patches that overlap with CD9-maxima crowds varies strongly, from none to several ones per membrane sheet. To get a better impression about the different shapes encountered, the lower row shows an anecdotal image of a membrane with several patches/crowds. Orange arrows point to 900 nm circles placed at locations with crowded maxima. (**B**) CD9- and pERM-clusters density. (**C**) Percentage of CD9-clusters plotted against distance to the next nearest pERM-cluster. The dashed vertical line marks 60 nm. Clusters with distances ≤ 60 are counted as overlapping clusters; the fractions are stated as percentages. (**D**) Percentage of CD9-maxima plotted against the number of their neighbored-maxima located in a 900 nm diameter circle. Dashed black line: Gaussian distribution fitted to control distribution (continuous black line). The dashed purple line retraces the ‘+ EWI-2’ distribution and is the sum of two Gaussian distributions: green (66% of the area below the dashed purple line) and orange (34%). Values are given as means ± SD (n = 3 biological replicates). (**E**, **F**) The intensity of a (**E**) CD9- and (**F**) pERM-maximum, measured in a 100 nm diameter circular ROI, plotted against the intensity in the respective other channel. For clarity, maxima intensities > 100 a.u. are not shown. Left, control; right, after EWI-2 elevation. R^2^ values refer to linear regression lines (see diagonal dotted lines) fitted to the data points and through the origin. Dotted lines parallel to the axes separate maxima with intensities below the noise level (≤ 2 a.u.). Percentage values refer to maxima above (magenta/green) and below (black) the noise level.

We next study ezrin–radixin–moesin proteins. These peripheral membrane proteins do not interact directly with the tetraspanins CD9 and CD151, but with the tetraspanin CD81 and the CD81/CD9 interaction partner EWI-2 (and EWI-F), thereby acting as a linker between TEMs and the actin cytoskeleton, regulating cell motility and polarity^27^. The activated, membrane-associated form is phosphorylated^29^, wherefore we use an antibody against phosphorylated ezrin–radixin–moesin proteins (pERM) (Fig. 4A and B).

As shown in Fig. 4C, the distance distribution peaks at 50 nm and is not as sharp as in Figs. 1C and 2C. 20% and 16% of CD9-clusters overlap with pERM-clusters in control and overexpressing cells, respectively (Fig. 4C). CD9-maxima and pERM-channel intensity do not significantly correlate; data points do not split up into two populations. The fraction of CD9-maxima positive for pERM is 27% in control and 20% in EWI-2 overexpressing cells (Fig. 4E), which is close to the fraction of overlapping clusters (for the fraction of pERM-maxima positive for CD9 see Fig. 4F). Importantly, a fraction of CD9-assemblies overlaps with/is close to activated ERM proteins.

In summary, CD9 concentrically overlaps/is proximal to CD81, EWI-2, CD44 and pERM, and CD151 overlaps with CD81. Hence, tetraspanin-maxima observed by microscopy represent multi-component assemblies, featuring the composition of assemblies identified in biochemical pull-down assays.

### Enrichment via cluster-agglomeration

Elevation of EWI-2 causes no substantial changes in cluster-density, inter-cluster distance, and inter-channel intensity-correlation. Yet, there is an effect on maxima, which can be described as maxima crowding (see arrows in Figs. 1A, 2A and 3A). For the quantification of crowding, for every maximum we determine the number of maxima in its neighborhood. As neighborhood area, we define a maximum-centered circle of 900 nm diameter, in which we count the additional maxima. Then, crowding is described by the percentage of maxima plotted against the number of neighbored-maxima; a normal distribution is expected if maxima are unorganized. Regarding CD9 and CD81, in control cells, we find a modestly right skewed distribution of CD9 and CD81 neighbored-maxima (see black lines in Fig. 1D and E), indicating that some maxima have more neighbors as expected under conditions of normal variability.

In this analysis, the peak positions of the neighbored-maxima distributions shift towards larger values with increasing maxima-densities. Elevation of EWI-2, although not changing the density of CD9- and CD81-clusters (Fig. 1B), yet strongly changes the neighbored CD81-maxima distribution. While in control cells the distribution peaks at ⁓ 4 neighbored maxima and is only modestly right skewed (Fig. 1E, black line), EWI-2-elevation diminishes the amplitude of the main peak and causes a broad shoulder around 10 – 15 neighbored-maxima (Fig. 1E, gray line). One interpretation is that a second population with several-fold more neighbored-maxima forms. In fact, the measured distribution (gray line in Fig. 1E) can be readily decomposed into two normal distributions, suggesting that 32% of maxima crowd stronger with on average ⁓ 12 neighbors (orange distribution in Fig. 1E), whereas the majority of maxima has ⁓ 3.5 neighbors (68%, green distribution in Fig. 1E). The effect of EWI-2 on the CD81-neighbored maxima distribution in Fig. 1E is reproduced in Fig. 2E, which only differs from Fig. 1 with respect to the identity of the counterstained tetraspanin (CD151 instead of CD9).

**Figure 5:**
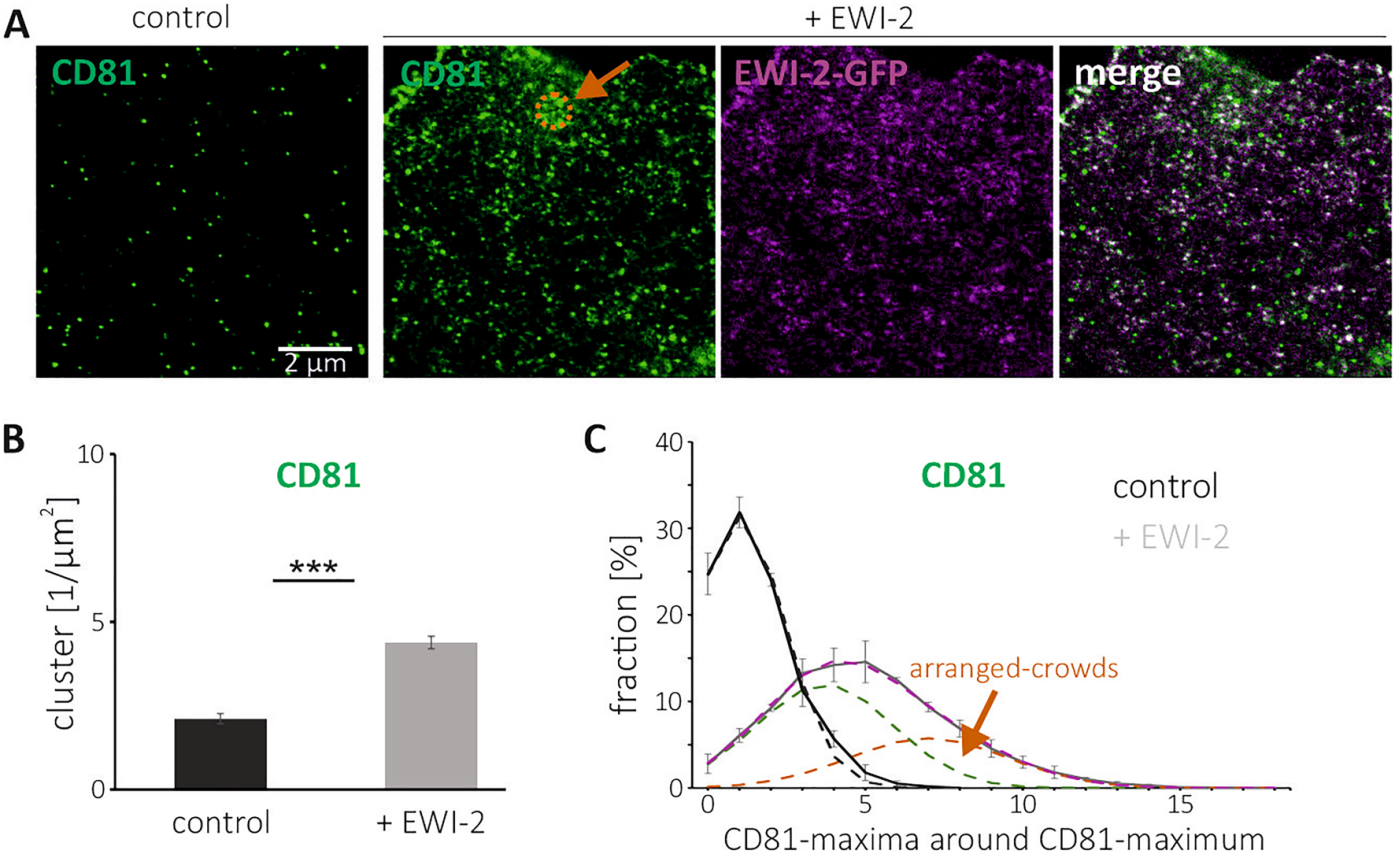
Cluster density and neighbored-maxima of CD81 in control cells and after EWI-2 elevation. (**A**) STED micrographs of membrane sheets generated from HaCaT cells (‘control’) or HaCaT cells overexpressing EWI-2-GFP (‘+ EWI-2’), fixed, and stained for CD81 (STAR RED). ‘Control’ was stained as well for F-actin (see legend of Fig. 1); membrane sheets from EWI-2-GFP overexpressing cells were stained for GFP with a nano-body coupled to Atto594. Orange circle marks a region with crowded CD81-maxima. (**B**) CD81-clusters density. (**C**) Percentage of CD81-maxima plotted against the number of their neighbored-maxima located in a 900 nm diameter circle. Dashed black line: Gaussian distribution fitted to control distribution (continuous black line). The dashed purple line retraces the ‘+ EWI-2’ distribution and is the sum of two Gaussian distributions: green (63% of the area below the dashed purple line) and orange (37%). Values are given as means ± SD (n = 3 biological replicates).

Next, we study again the effect of EWI-2 on CD81 crowding, this time visualizing CD81 together with overexpressed EWI-2-GFP (Fig. 5A). Because the GFP-chromophore is not suitable for STED-microscopy, for EWI-2-GFP visualization we employ a GFP-nanobody coupled to Atto594 (Fig. 5A). As shown in Fig. 5B, under elevated EWI-2, the CD81-cluster density is similar as in Figs. 1B and 2B, but in the control, it is markedly lower. We speculate that, depending on the expression level of other tetraspanin web components, EWI-2 elevation may promote cell-surface trafficking of CD81. In any case, at elevated EWI-2, we observe a right skewed distribution of neighbored CD81-maxima. Decomposition into two normal distributions suggests that 37% of the CD81-maxima crowd stronger (Fig. 5C). The crowded fraction is even larger than in Fig. 1E (32%), but peaks at a lower neighbored-maxima density, precluding a clear visual separation of the two distributions.

Next, we examined the effect of EWI-2 overexpression in another human cell line, Jurkat T cells. As shown in Supplementary Fig. 7, EWI-2 overexpression has no effect on the CD9-cluster density, but more than doubles the CD81-cluster density (Supplementary Fig. 7B). Occasionally, there are areas with strongly agglomerated maxima. In contrast to control cells, where more than half of the maxima has not a single neighbored-maximum, maxima in EWI-2 overexpressing cells have up to ⁓ 10 neighbored-maxima (Supplementary Fig. 7C).

We observe no effect on CD151 crowding (Fig. 2D). The EWI-2 effect on CD9 is unclear. While the neighbored-maxima distribution is unaltered in Figs. 1D and 3D, in Fig. 4D, we observe a trend towards a slightly broader distribution with a reduced amplitude, a main peak shifted towards less neighbored maxima, and a shoulder at higher neighbored-maxima values (compare black ‘control’ and gray ‘ + EWI-2’ lines). The CD9-distribution can be decomposed into two normal distributions peaking at ⁓ 7.5 (green) and ⁓ 10 (orange) neighbored-maxima (Fig. 4D; green + orange Gaussians = purple Gaussian). This is similar to the behavior of CD81 in Fig. 1E.

We speculate that two populations underlie the modest right skewed distribution in control cells. One of randomly scattered maxima and one of agglomerated maxima. EWI-2 promotes agglomeration; the more EWI-2 increases the fraction of agglomerating maxima, the more right-skewed the distribution becomes. In extreme cases, the distribution splits up into a two-peak distribution. After un-mixing the neighbored maxima distribution into two Gaussians, the neighbored-maxima size distribution of the more crowded maxima is described by the Gaussian peaking at higher neighbored-maxima number. To differentiate agglomerated- from randomly-crowded maxima, we refer to agglomerated maxima as arranged-crowds (ACs; see orange arrows in Figs. 1E, 2E, 3E, 4D and 5C).

If EWI-2 triggers the formation of ACs, it may enrich in ACs as well, triggering its own agglomeration. In control cells, the EWI-2 neighbored maxima-distribution is nearly normal (Fig. 3E; compare black line of raw data to dashed line of the Gaussian fit). After EWI-2 elevation, because of the doubling of EWI-2 clusters (Fig. 3B), the peak of the distribution shifts towards larger values. The distribution also becomes right skewed (gray line in Fig. 3E). Decomposition yields normal distributions peaking at ~ 4 and ~ 9 neighbored-EWI-2-maxima (green and orange dashed lines; purple = green + orange). Hence, as speculated above, EWI-2 overexpression generates its own agglomeration into ACs.

In conclusion, EWI-2 elevation promotes the agglomeration of CD81-maxima and its own maxima into arranged-crowds. Regarding CD151 there is no agglomeration effect, and the behavior of CD9 is unclear.

### Features of ACs

ACs are microscopically-defined local enrichments of tetraspanin-assemblies. Like TEMs, they could be functional architectures. As actin-dynamics are associated with many tetraspanin functions, actin may be required for AC-formation. For testing this hypothesis, we overexpressed EWI-2 to promote ACs, and one hour prior to fixation, 25 µM latrunculin was added to inhibit actin-polymerization. No effect on the distribution of neighbored-maxima of EWI-2 is observed (Supplementary Fig. 8C). Hence, the integrity of EWI-2-AC is independent from actin-polymerization. We next wondered whether ACs need actin-dynamics for their generation. Latrunculin B was added earlier, already 8 h after transfection, and kept in the culture for 16 h, until fixation. Again, no effect was observed (Supplementary Fig. 8C).

**Figure 6:**
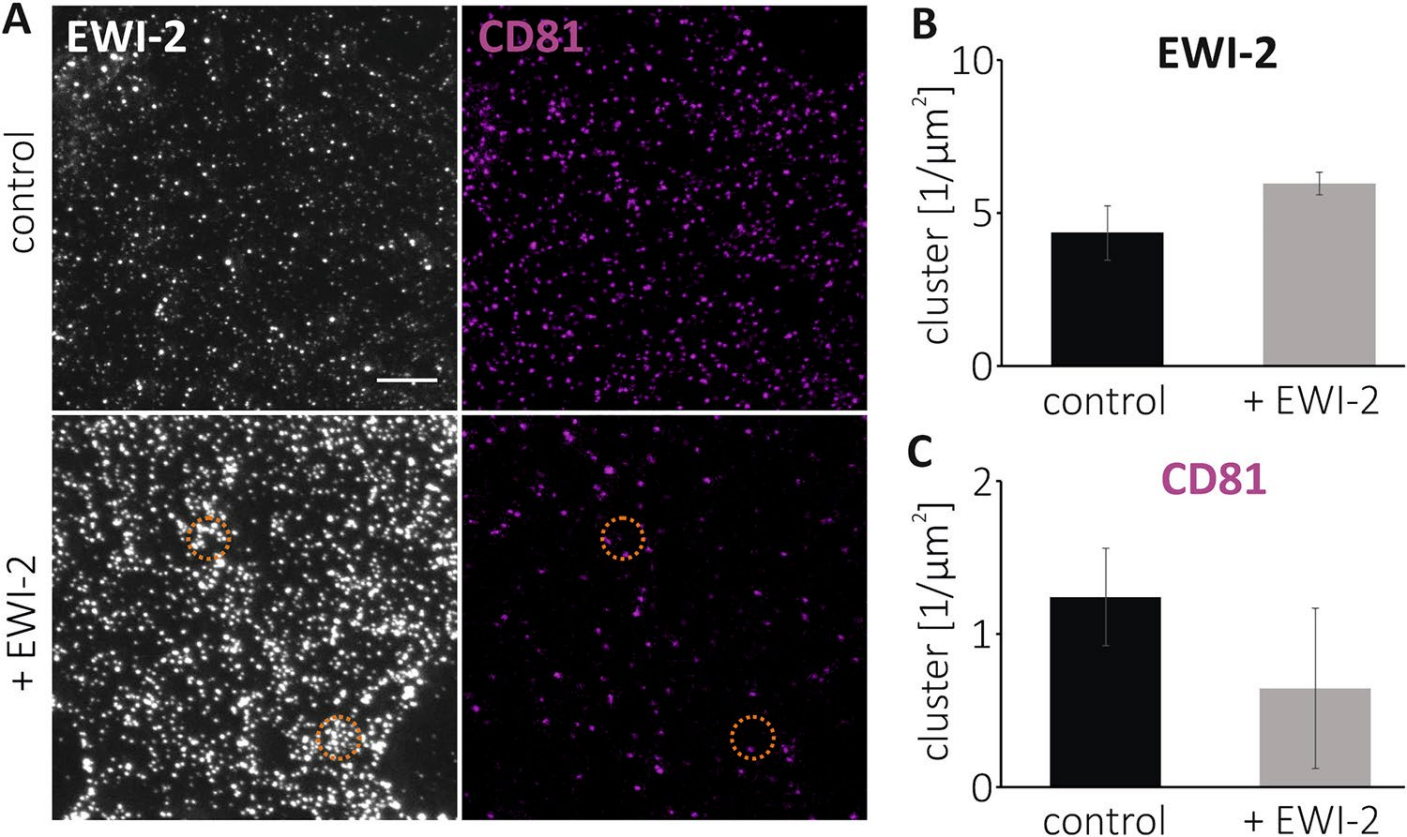
A monoclonal antibody against the δ-loop of CD81 detects less CD81-clusters. (**A**) STED micrographs of membrane sheets generated from HaCaT cells (‘control’) or HaCaT cells overexpressing EWI-2-GFP (‘+ EWI-2’), fixed, and stained for CD81 (Alexa594) and EWI-2 (STAR RED). Orange circle marks regions with crowded maxima detected only in the EWI-2-channel. (**B**) EWI-2- and (**C**) CD81-clusters density. Values are given as means ± SD (n = 3 biological replicates).

In CD81-ACs, CD81 maxima have agglomerated into a structure that likely is rich in CD81-oligomers/CD81-interaction partners. We speculate that CD81 in ACs is stronger involved in interactions when compared to CD81 outside of ACs. For testing this hypothesis, we used a monoclonal antibody recognizing the δ-loop of CD81, required for CD81 homo-dimerization^30^. If the loop plays a role in AC formation, CD81 in ACs may be difficult to detect due to epitope shielding after complex-formation. In control cells, the monoclonal antibody tends to detect much less CD81-clusters (Fig. 6C) when compared to the polyclonal antibody used in Figs. 1 and 2. Elevation of EWI-2 diminishes the number of CD81 clusters (Fig. 6C), different from the polyclonal antibody, for which we observe either no EWI-2 effect (Figs. 1B and 2B), or even a strong increase in CD81-cluster density (Fig. 5B). Altogether, when compared to the polyclonal antibody, after EWI-2 elevation (Fig. 6B) the monoclonal antibody detects roughly one order of magnitude less CD81-clusters (compare Fig. 6C to Figs. 1B, 2B and 5B). The undetected maxima preferentially may be those in ACs, as no CD81-maxima crowds are seen in areas of crowded EWI-2 (see circles in Fig. 6A). This observation supports the view that arrangement of CD81-maxima into ACs is accompanied by a stronger involvement in interactions and as a result, the δ-loop of CD81 becomes shielded and is no longer detectable by the antibody. However, the monoclonal antibody detects CD81 also less efficient in areas outside of ACs.

In conclusion, tetraspanin-maxima represent plasmalemmal entities used as building blocks for EWI-2-driven agglomeration into extended, densely crowded tetraspanin-architectures. These architectures contain as well EWI-2 and pERM. Because ACs are plasmalemmal areas of tetraspanin enrichment, they are a microscopic complement to biochemically defined TEMs.

## Discussion

### Relationship between protein copy number and signal-intensity

In fluorescence microscopy, we generate a contrast between a protein of interest and all other cellular components. Ideally, recorded fluorescence-intensity is directly proportional to the copy number of the detected protein. For that, each protein must be labelled with equal amounts of fluorescent moieties, and each fluorophore must be detected with equal efficiency. This is impossible, in particular, if proteins are densely packed. For instance, in oligomers of GFP-labelled proteins, GFP does self-quench^31,32^. Strongly quenched oligomeric molecules and normally fluorescent non-oligomeric molecules result in a distortion of intensity-values across the image; intensity-values are no longer proportional to protein copy number.

The same occurs with antibodies, provided their physical size is larger than the epitope-distances in the oligomerized proteins. Due to steric reasons, an antibody cannot bind to every protein. Additionally, epitopes may remain undetected because after complex formation they become masked. Therefore, detection eventually depends as well on the proteins’ expression level (that may regulate its oligomeric state) and the abundancy of its binding partner. Alternatively, some tetraspanins may adopt a different conformation, or do not mature properly into a conformation detected by the antibody. In one micrograph, we may encounter areas where the signal is linearly related to copy number, exponentially saturating, or not detectable at all.

In this study, we analyze the lateral distribution of assemblies, finding that they form ACs. In comparison to a polyclonal antibody, a monoclonal antibody detects less CD81-clusters, in particular after EWI-2 elevation (Fig. 6C), and in these images we do not observe crowded CD81-maxima (Fig. 6A). Thus, not every antibody is suitable for detecting ACs. We do not know whether our monoclonal CD9 antibody detects CD9-ACs. Only in one out of three experiments a possibly weak EWI-2 effect on the distribution of neighbored-CD9-maxima is observed (in Fig. 4D, but not in Figs. 1D and 3D).

In any case, epitope-masking, if it occurs, does not preclude the detection of CD81 and EWI-2 in ACs. Therefore, despite of technical limitations, we conclude that EWI-2-elevation causes the agglomeration of CD81 and its interaction partner EWI-2.

Analyzing inter-channel maxima-intensity correlations, after fitting of a regression line through the origin there are no significant correlations. Yet, CD9-maxima are positive for/overlap with CD81 (Fig. 1C and F), EWI-2 (Fig. 3C and F), CD44 (Supplementary Fig. 6C) and pERM (Fig. 4C and E), CD81-maxima are positive for CD9 (Fig. 1G), CD151 maxima are positive for/overlap with CD81 (Fig. 2C and F), and CD81-maxima are positive for CD151 (Fig. 2G). Moreover, we can differentiate between two populations of CD81-maxima differing in their CD81:CD9 ratio (see orange boxes in Fig. 1G). We assume that shielding effects and other technical limitations influence the result, maybe even disguise possible positive correlations. For instance, if there is an accumulation of many CD81 and few CD9 molecules, decoration of the assembly with CD81 antibodies may preclude binding of any further CD9 antibody. Alternatively, it is possible that the composition of the assemblies is governed by mechanisms that do not follow biochemical equilibriums. Albeit the observation of different populations of assemblies is interesting and deserves further investigation, in this study, it is a side aspect. Our main finding that microscopically observed assemblies contain more than one protein remains not obscured by epitope masking. Epitope-shielding will not create but decrease overlap. Actually, we believe we underestimate overlap in some of the stainings. For instance, considering that in biochemical experiments 70% of CD9 associates with EWI-2 in embryonic kidney cells^25^, the fraction of CD9 clusters overlapping with EWI-2 clusters is surprisingly low (only 11% in control and 22% in EWI-2 overexpressing cells).

### Co-assembly of CD9 with tetraspanins and TEM components

Almost 30 years ago, biochemical dissection of tetraspanin-assemblies uncovered a network of interactions between tetraspanins and their partner proteins. Analyzing tetraspanins and their partners by microscopy should uncover the spatial parameters of the tetraspanin web.

If tetraspanins organize in multimeric-assemblies, as suggested by biochemistry, in fluorescence microscopy, signals of different tetraspanins should overlap. A previous study in immune cells analyzed the distribution of the tetraspanins CD37, CD53, CD81 and CD82 in comparison to the GPI-protein CD55 (another ‘cluster of differentiation’ molecule that, however, is not a tetraspanin), a protein excluded from the tetraspanin-web^24^. Like in our study, tetraspanins were visualized by immunolabeling of membrane sheets followed by STED microscopy analysis. Tetraspanins concentrated in clusters with a size of ⁓ 120 nm, and CD53-clusters were not closer to CD37-, CD81- and CD82-clusters when compared to clusters of the non-tetraspanin web marker CD55^24^. Moreover, CD81- and CD82-clusters did not concentrically overlap with CD37-clusters, but were within a 100 nm distance, indicating partial co-clustering. The authors concluded that, at least regarding CD53, in contrast to the current dogma of a TEM, individual members of the tetraspanin family are present in clusters that are largely devoid of other members of the tetraspanin family^24^.

In our study, we examined the ubiquitously expressed tetraspanins CD9, CD81, and CD151. CD9 and CD81 exhibit an approximately 60% sequence similarity and have the same overall structure^16^. Moreover, they share EWI-2 as a binding partner^25^. The similarity may explain why they readily co-assemble, as indicated by 40% of CD9-clusters concentrically overlapping with CD81-clusters (Fig. 1C, control). However, clusters of a different tetraspanin, as CD151, overlap with CD81-clusters equally well (Fig. 2C). In any case, microscopy identifies co-assemblies between CD9 and CD81 (Fig. 1) and between CD151 and CD81 (Fig. 2). Moreover, microscopy shows that tetraspanin-assemblies contain as well tetraspanin interaction partners, albeit at this point we have only tested for EWI-2 (Fig. 3), pERM (Fig. 4), and CD44 (Supplementary Fig. 6).

In principle, microscopic assemblies are indistinguishable from previously identified biochemical assemblies. Therefore, we propose that multimeric-tetraspanin assemblies, known from biochemistry, are equivalent to the maxima/clusters we here characterized by microscopy. We find large heterogeneity in assembly composition and local crowding. Maxima scatter largely unorganized across the cell surface, but at some sites they agglomerate into ACs. In ACs, tetraspanin nanodomains are more abundant in the membrane than outside of ACs, which makes them to microscopically defined tetraspanin-nanodomain enriched areas.

### Tetraspanin assemblies constitute building blocks for tetraspanin-enriched domains

**Figure 7:**
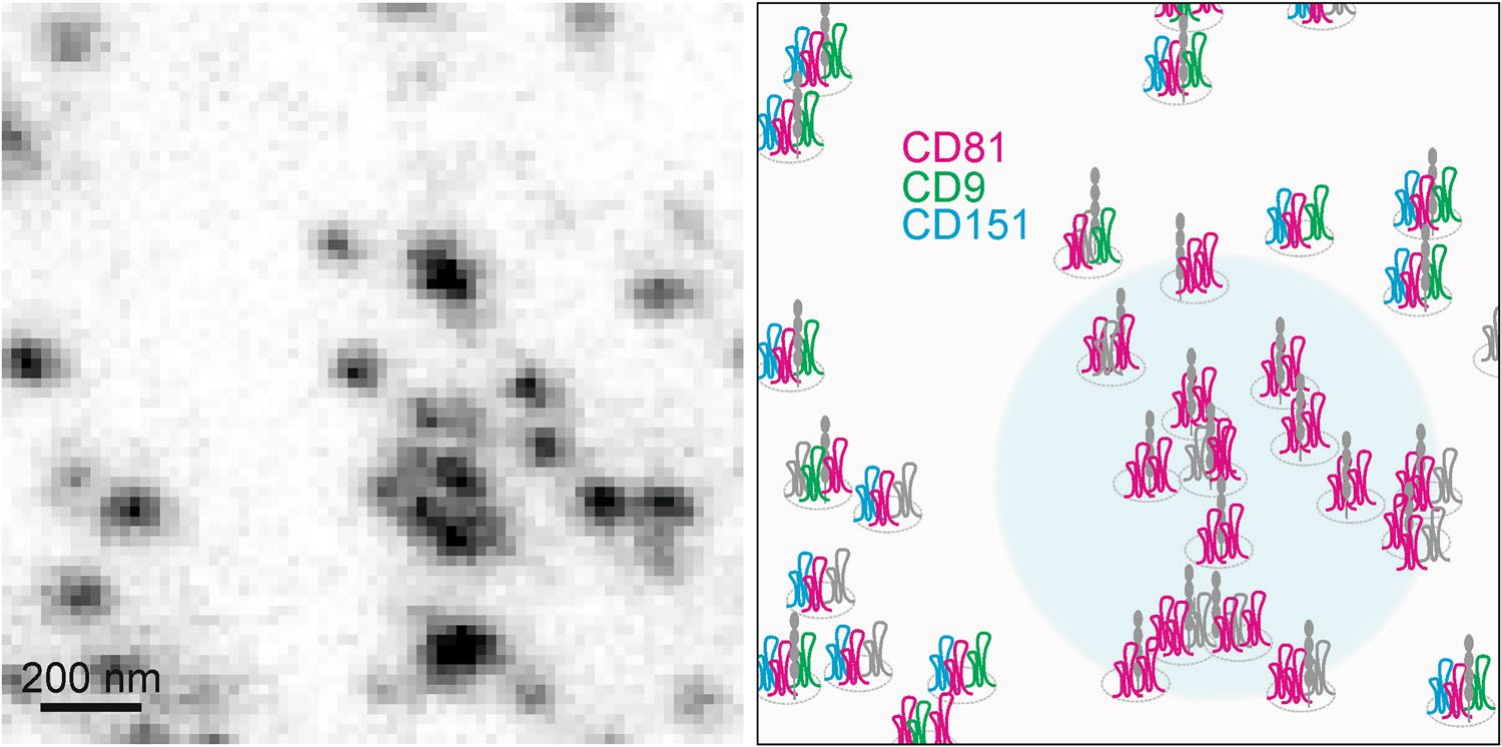
*Tetraspanin assemblies and tetraspanin-nanodomain enriched areas.* Based on the imaging data, we draw two biologically relevant conclusions. First, maxima (black dots in left image) arise from assemblies (marked by small circles in the right panel) frequently constituted of more than one tetraspanin family member and they may contain tetraspanin interaction partners as EWI-2 (gray molecule) as well. The copy numbers are highly variable. For clarity, we draw per assembly only one icon per tetraspanin family member. Other tetraspanins (gray tetraspanin icons) than CD81, CD9 and CD151 may be present as well. Second, EWI-2 elevation arranges the assemblies into arranged crowds that represent tetraspanin-nanodomain enriched areas. We also draw a technically relevant conclusion. As tetraspanins are small proteins deeply rooted into molecular networks, antibodies cannot reach the epitopes of all tetraspanin molecules present in the cell membrane. Hence, we underestimate overlap, and may oversee e.g. CD151 and CD9 crowding. This, however, does not affect the two main biological conclusions. Left, image from the experimental series of Fig. 1, showing an arranged CD81-crowd with more than 10 maxima in the lower right. The filled large circle (right panel) has the size of the 900 nm search radius used for counting neighbored-maxima.

In control cells, the distribution of neighbored-maxima is roughly normal (see e.g. Fig. 1D and E) suggesting that the majority of CD9- and CD81-maxima scatters unorganized across the cell membrane. Upon elevation of EWI-2, maxima agglomerate into ACs (see Figs. 1E, 2E, 3E, 4D, and 5C) that are architectures of irregular shape and size, covering areas in the square-micrometer range. Do CD81- and CD9-maxima agglomerate to the same extent? If CD9- and CD81-assemblies contribute equally to the formation of ACs, the neighbored-maxima distributions of CD81 and CD9 should change in a similar way. However, we see a change only of CD81-maxima. Due to possible antibody-shielding effects, our CD9 antibody may not detect agglomerated CD9-maxima. On the other hand, CD81-agglomeration may involve preferentially the population of CD81-only-maxima (see model in Fig. 7). In any case, EWI-2 promotes the formation of maxima-crowding and of tetraspanin-nanodomain enriched areas. The microscopic EWI-2-agglomeration effect parallels biochemical data, showing that elevation of EWI-2 increases the extent of complexes between CD81 molecules and CD81 and its interaction partner α4β1^33^.

In ACs, single tetraspanin assemblies maintain their integrity; the force driving agglomeration is not capable of fusing several multimeric-tetraspanin assemblies into a single super-large assembly. Moreover, for EWI-2-agglomeration, actin polymerization is not required. Aside from tetraspanins, also EWI-2 forms ACs (Fig. 3). ACs may differ between cell types. In Jurkat T cells, for instance, it appears that the agglomeration effect is even more prominent (Supplementary Fig. 7).

### Outlook

Almost three decades ago, biochemical dissection of tetraspanin interactions lead to the concept of an interaction network between tetraspanins and their interaction partners – the tetraspanin web. However, tetraspanins are still puzzling proteins. We do not understand how they are capable of playing roles in that many cellular processes, and why a tetraspanin in a different cellular context shows an opposite effect.

Here, we shed light on the lateral organization of tetraspanins, uncovering different levels of organization. Different tetraspanin family members and interaction partners form assemblies that are capable to agglomerate into tetraspanin enriched domains. This conceptual framework provides a link between biochemistry and microscopy. Combining both methodologies will enable us to unravel the functional mechanisms of this enigmatic protein family.

## Material/methods

### Antibodies and plasmid

The following antibodies were used in immunostaining experiments. Primary antibodies: for EWI-2 a goat polyclonal-antibody (AF3117; R&D Systems, Minneapolis, USA), for CD81 a rabbit polyclonal antibody (sc-9158 H-121, Santa Cruz, Texas, USA) and a mouse monoclonal antibody (sc-7636 1.3.3.22, Santa Cruz), for CD9 a mouse monoclonal antibody (MM2-57 CBL162, Merck, Darmstadt, Germany), for pERM a rabbit monoclonal antibody (mAB 3726T, Cell Signaling Technology, Massachusetts, USA), for CD151 a mouse monoclonal antibody (Biorad MCA1856GA) and for CD44 a rabbit polyclonal antibody (15675-1-AP, ProteinTech, Rosemont, USA).

As secondary antibodies we used: STAR RED coupled to donkey-anti-goat (STRED-1055-500UG, Abberior, Göttingen, Germany), goat-anti-rabbit (STRED-1002-500UG, Abberior), and goat-anti-mouse (STRED-1001-500UG, Abberior). AlexaFluor™ 594 coupled to donkey-anti-mouse (A21203, Invitrogen™, Carlsbad, USA), donkey-anti-goat (A11058, Invitrogen™) and donkey-anti-rabbit (A21207, Invitrogen™). Moreover, we used nanobodies GFP booster Atto488 (Alpaka nanobody Gba488-100, ChromoTek, Rosemont, USA) and GFP booster Atto594 (Alpaka nanobody Gba594-100, ChromoTek).

### Cloning of EWI-2-GFP

The C-terminus of EWI-2 (NM_001320247) was fused to monomeric GFP^30^, using the expression vector pEGFP-C1. For cloning of EWI-2-GFP, the NEBuilder Hifi DNA Assembly® cloning kit was used (E5520S, New England Biolabs). The template for the EWI-2 gene was the EWI-2 RFP construct described in Ref.^30^. The monomeric GFP was amplified from CD81-GFP also described in Ref.^30^. We used primers up to 35 bp long with 5´overhangs of 15 bp. For the linearized vector with EWI-2 we used: forward gacgagctgtacaag-taactgatcataatcagcc; reverse gcccttgctcaccat-ccgttttcgaagcctcttc; For GFP we used: forward aggcttcgaaaacgg-atggtgagcaagggcgagg; reverse gattatgatcagtta-cttgtacagctcgtccatgc. First, the vector with EWI-2 was amplified via PCR yielding a linearized vector. GFP was amplified by PCR yielding GFP with EWI-2/vector overhangs. Methylated DNA was removed by DpnI treatment. EWI-2-GFP fusion was realized by the assembly reaction at a vector:insert ratio of 1:2. The construct was verified by sequencing.

### Cell culture

HaCaT cells (Cell Lines Services, Eppelheim, Germany) were cultured in high glucose DMEM (Gibco® 61965-026) medium supplemented with 10% FCS (PAN Biotech, cat# P30-3031, Aidenbach, Germany) and 1% Penicillin/Streptomycin (10,000 U/ml Penicillin, 10 mg/ml Streptomycin; PAN Biotech, cat# P06-07100) at 37 °C with 5% CO_2_. Cells were transfected using the NEON™ Transfection Kit (ThermoFisher Scientific, Waltham, USA) according to the user manual employing the specific setting for HaCaT cells (1600 V; 10 ms; 3 times). Per transfection 2.5 × 10^6^ cells and 12.5 μg of EWI-2-GFP plasmid (purified using the NucleoBond® XtraMidi Kit (Ref: 740410.100 Macherey–Nagel, Düren, Germany)) were used. Control cells were transfected using instead of plasmid equal volumes of ddH_2_0. Approximately 300,000 cells were plated per poly-L-lysine coated (100 µg/ml) glass coverslips (25 mm diameter) and incubated around 24 h prior to fixation.

HepG2 cells (Cell Line Services, Eppelheim, Germany, cat# 300198) were cultured in MEM Eagle Medium (PAN Biotech, cat# P04-08509), supplemented with 10% fetal bovine serum (PAN Biotech, cat# P30-3031), 1% 200 mM stable glutamine (PAN Biotech, cat# P04-82100) and 1% penicillin–streptomycin (PAN Biotech, cat# P06-07100) at 37 °C with 5% CO_2_.

Jurkat E6.1 cells (ECACC, purchased from Sigma-Aldrich Germany, # 88042803) were cultured in RPMI-1640 (#21875-034, Gibco, UK) medium supplemented with 10% fetal bovine serum (PAN Biotech, #P30-3031), 1% penicillin–streptomycin (PAN Biotech, #P06- 07100) at 37 °C with 5% CO_2_. Cells were transfected using the Gene pulser Xcell electroporation system (Bio-Rad, Hercules, CA, USA). In brief, 10^7^ cells were resuspended in 800 μl Cytomix (120 mM KCl, 10 mM KH_2_PO_4_, 10 mM K_2_HPO_4_, 0.15 mM CaCl_2_, 2 mM EGTA, 5 mM MgCl_2_, 25 mM HEPES–KOH, pH 7.6) and mixed with 30 μg plasmid. The electroporation was performed in a 4 mm electroporation cuvette using the settings: Exponential protocol, 250 V, 1500 µF and infinite Ω. Afterwards, cells were incubated for 2 days in culture. Then, approximately 300,000 cells were plated in Ringer solution (130 mM NaCl, 4 mM KCl, 1 mM CaCl_2_, 1 mM MgCl_2_, 48 mM D( +) glucose, 10 mM HEPES–NAOH, pH 7.4) onto poly-L-lysine coated glass coverslips and incubated for 20 min prior to membrane sheet generation.

Latrunculin B (Cay10010631-1, Biomol, Hamburg, Germany) was used from a 63 mM stock solution in ethanol. For latrunculin B treatments, cells were transfected with EWI-2-GFP and incubated either 8 h after transfection for another 16 h with latrunculin B, or after 23 h after transfection for 1 h with latrunculin B. To this end, medium was replaced with antibiotic-free medium containing 25 μM latrunculin B.

### Immunostaining

For membrane sheet generation, cells on a coverslip were placed in ice cold sonication buffer (120 mM KGlu, 20 mM KAc, 10 mM EGTA, 20 mM HEPES, pH 7.2) at a distance to the sonication tip of about 1–2 mm. Then, moving around the coverslip about 10 times, we applied for HaCaT cells at different locations a 100 ms ultrasound pulse at 100% power (employing a Bandelin Sonoplus GM2070 sonifier). For HepG2 and Jurkat T cells we applied only one pulse at 80% power and 10% power, respectively. The sample was fixed in 4% PFA (in PBS; 137 mM NaCl, 2.7 mM KCl, 1.76 mM KH_2_PO_4_, 10 mM Na_2_HPO_4_, pH7.4) for 30 min, followed by PFA quenching with 50 mM NH_4_Cl in PBS for 20 min. Membrane sheets were permeabilized for 1 min using 0.2% TritionX-100 in PBS, and blocked for 1 h in 3% BSA-PBS. Primary antibodies were diluted 1:200 (1:500 for CD9) in 3% BSA-PBS and incubated with the membrane sheets for 2 h. Coverslips were washed, and incubated with secondary antibodies diluted 1:200 in 3% BSA-PBS (1:10,000 when using secondary antibodies in CD44 stainings) for 1h in the dark. For controls, the secondary antibody solution contained Phalloidin488 (at 1:1000 dilution; ab176753, Abcam). When membrane sheets from cells overexpressing EWI-2-GFP were studied, the secondary antibody solution contained GFP booster Atto488 (at 1:200 dilution); for Fig. 5 we used GFP booster Atto594 (at 1:200 dilution). After washing, coverslips were mounted in mounting medium (ProLong™ Gold antifade reagent, Invitrogen, P36930) and sealed with nail polish.

### Microscopy

The samples were examined by confocal and STED microscopy employing a 4-channel STED microscope from Abberior Instruments (available at the LIMES institute imaging facility, Bonn, Germany). The microscope is based on an Olympus IX83 confocal microscope equipped with an UPlanSApo 100x (1.4 NA) objective (Olympus, Tokyo, Japan). For STED microscopy, we used 561 nm (0.3 mW at 40%; detected at 580–630 nm (red channel); 5 line steps) and 640 nm (1 mW at 50% (20% for CD44 staining); detected at 650–720 nm (long red channel); 5 line steps) lasers for excitation, and a 775 nm laser (1.2 W at 50%) for depletion. When analyzing double stainings using Alexa594 (excited with 561 nm) together with STAR RED (excited with 640 nm), we observed significant co-excitation of Alexa 594 when the 640 nm laser was employed at a power above 20%. Therefore, at 50% laser power, prior to image analysis we subtracted from the long-red-channel-image 50% of the red-channel-image, which results in a small overcorrection. For confocal microscopy, we used a 485 nm laser (1 mW at 20%; detected at 500–550 nm; 2 line steps), a 561 nm laser (at 30%; 2 line steps) and a 647 nm laser (at 20%; 1 line step). The pixel size was 20 nm.

CD9 was stained with the mouse monoclonal antibody (see above) in combination with donkey-anti-mouse-Alexa Fluor 594. CD81 was stained (except in the epitope masking experiment where we used a mouse monoclonal antibody) with the rabbit polyclonal antibody in combination with goat-anti-rabbit-STAR RED (except when double stained with EWI-2, here we used donkey-anti-rabbit-Alexa594). We screened for membrane sheets in the green channel, using as reference the Phalloidin488 staining on non-transfected cells, and the GFP fluorescence (amplified with GFP-nanobodies) of transfected cells. Then confocal micrographs were taken, followed by taking STED micrographs.

### Image analysis

Micrographs were analyzed using Fiji ImageJ in combination with a macro, essentially as previously described^34^. In brief, noise was reduced by a Gaussian blur (σ = 0.5). Then, local maxima were detected using the ImageJ function ‘Find maxima’ (noise tolerance 4 for CD9/CD81/CD151; 8 for CD44, 5 for pERM, 6 for EWI-2), yielding maxima positions defined as pixel positions.

For measuring maxima intensity, a ROI with a diameter of 100 nm (5 pixel-diameter; the central pixel is the maxima position) was used to measure the intensity of the maximum, and the intensity in the respective other channel. Intensity values were background corrected using a value determined with a ROI placed next to the membrane sheet.

For measuring the shortest distance between clusters, initially, a linescan analysis was performed in order to select clusters. To this end, on each maximum, either a horizontal or a vertical linescan was placed onto the maximum position (31 pixels long × 3 pixels width). A Gaussian function was fitted to the intensity distribution measured by the linescans. Maxima were rated as clusters if they have a fit quality of R^2^ > 0.8 and a centered peak (in the middle third of the linescan). The above-mentioned 100 nm ROI at the maxima position was used to measure the center of mass of fluorescence, yielding the cluster-positions at sub-pixel resolution. The sub-pixel resolution positions were further used to determine the shortest distance between clusters.

For measuring the neighbored maxima, the above-mentioned 100 nm ROI was enlarged to a 900 nm circular ROI, and placed onto the maxima positions from the ‘Find maxima’ function (see above). The number of maxima within the circular ROI was counted (employing the same noise tolerance as used for identifying the maxima) and the value of one was subtracted to account for the fact that the central maximum is not counted as its own neighbor.

In single Figures (one set of experiment), the same channels are shown at the same scaling (between Figures (different sets of experiments), images are shown at different scalings). Lookup-tables were adjusted in ImageJ, and images then exported as JPEGs into Corel-Draw.

### Statistics

Data was tested for significance with a two-sided, unpaired student´s *t* test with significance * = p < 0.05, ** = p < 0.01 and *** = p < 0.001. Data sets were based on three biological replicates, including, if not stated otherwise, 14–20 membrane sheets per replicate.

### Supplementary Information

Supplementary Figures.

## Data Availability

On reasonable request, raw data/from raw data generated data sets and sequencing data of the current study are available from the corresponding author.
