# Supplementary material for: Microscopic clusters feature the composition of biochemical tetraspanin-assemblies and constitute building-blocks of tetraspanin enriched domains
Source: Sci Rep. 2024 Jan 24;14:2093. doi: 10.1038/s41598-024-52615-1 (PMC10808221; doi:10.1038/s41598-024-52615-1)
Supplement: Supplementary file 1 — Supplementary Figures. [file 41598_2024_52615_MOESM1_ESM.docx]

## Supplementary Information

## Microscopic clusters feature the composition of biochemical tetraspanin-assemblies and constitute building-blocks of tetraspanin enriched domains

Sara C. Schmidt^1^, Annika Massenberg^1^, Yahya Homsi^1^, Dominik Sons^1^, and Thorsten Lang^1, *^

^1^University of Bonn, Faculty of Mathematics and Natural Sciences, Life & Medical Sciences (LIMES) Institute, Carl-Troll-Straße 31, 53115 Bonn, Germany

*Correspondence should be addressed to Thorsten Lang (thorsten.lang@uni-bonn.de)

Supplementary Figures 1 - 8


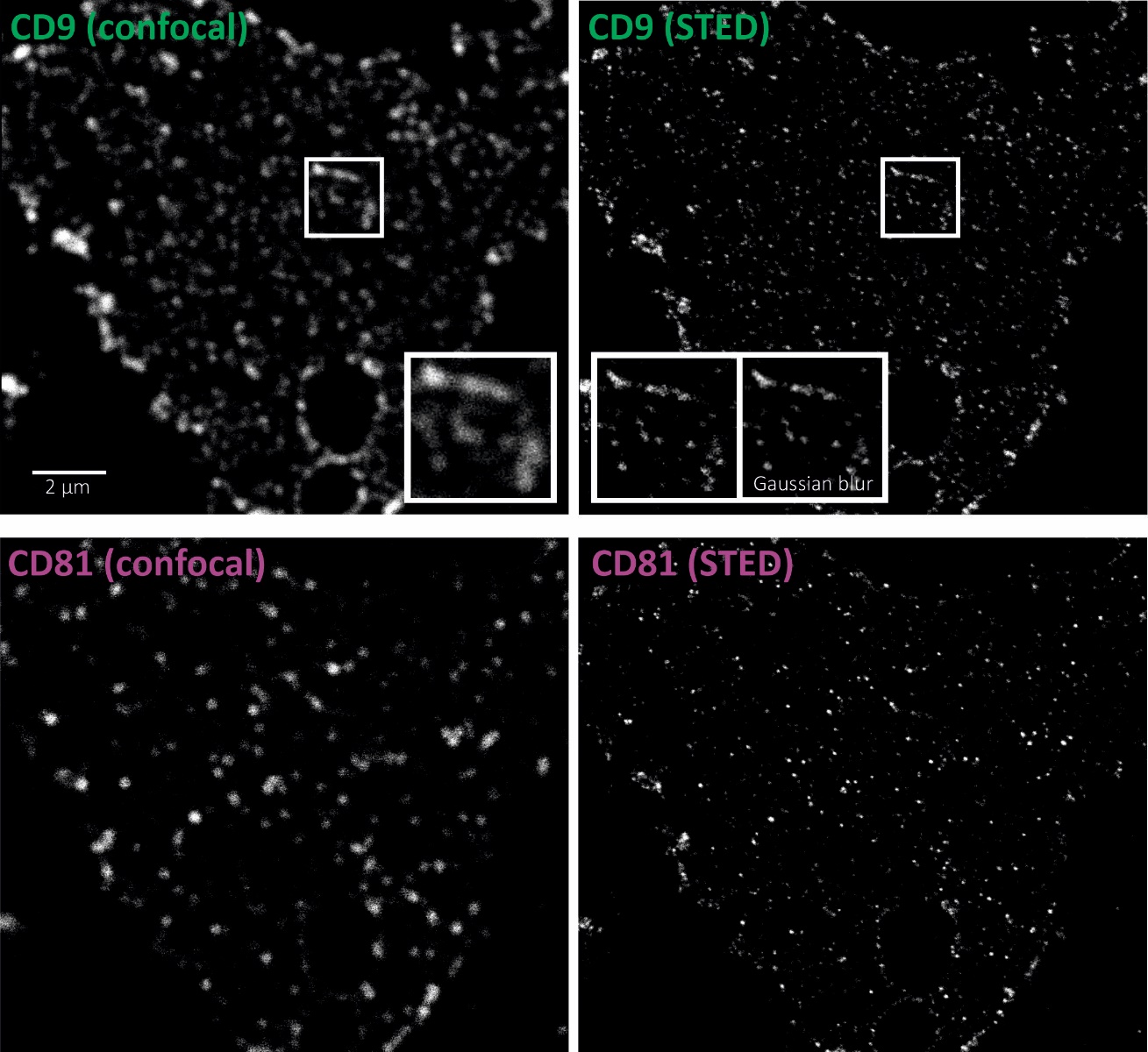


**Supplementary Figure 1.** *Comparison of confocal and STED microscopy.* Panels show raw images from which the panels of Figure 1A (control) were generated. They show a HaCaT cell membrane sheet immunostained for CD9 (top) and CD81 (down). Left, confocal images, right STED-micrographs. For better comparison, we show magnified views of the CD9 confocal- and STED-channel next to each other. Prior to image analysis, the ImageJ Gaussian blur filter was applied (compare the two magnified views of the CD9 STED-micrograph). Throughout all figures, we show the images on which the analysis was performed.


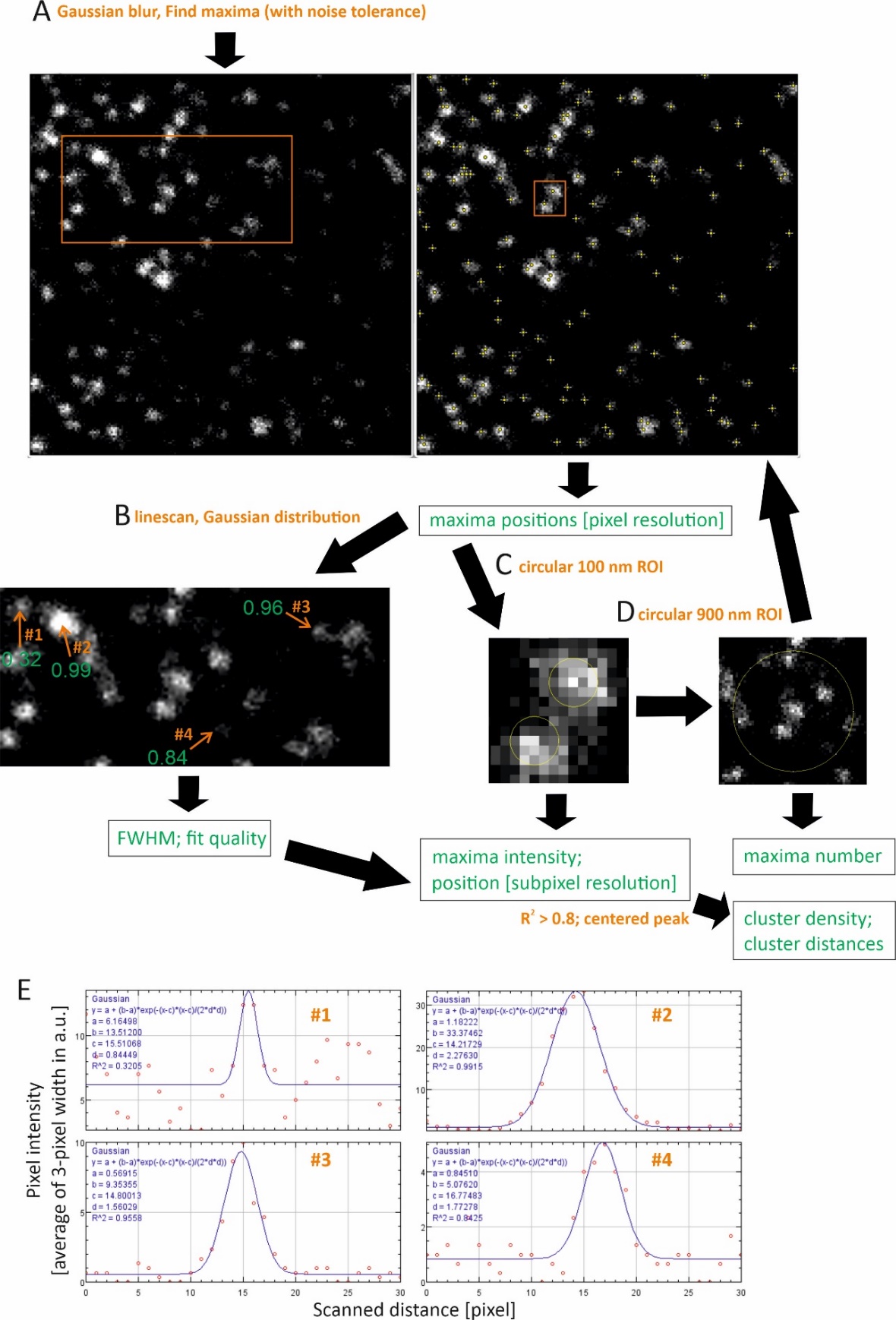


**Supplementary Figure 2.** *Scheme of image analysis.* For image analysis, the program ImageJ was used. (A) To reduce noise, a ‘Gaussian blur’ filter (σ = 0.5) is applied to the raw images (shown are filtered images; for the filter effect see Supplementary Figure 1). Then, using the ‘Find maxima’ function, maxima positions are determined with a specific noise tolerance. Is the noise tolerance too high, authentic maxima are lost; is it too low, false maxima (noise) are included. The best compromise depends on the characteristics of the staining intensity and must be found out manually. Throughout all analyses, we use a noise tolerance of 4 for CD9/CD81 (monoclonal antibody)/CD81 (polyclonal antibody)/CD151 (monoclonal antibody), 5 for pERM, 6 for EWI‑2, and 8 for CD44. In the right image of (A), yellow crosses mark maxima identified by this approach using a noise tolerance of 4 on a CD81 polyclonal antibody image. The unit of maxima positions is pixel coordinates (integer values). (B) A 31-pixel long and 3-pixel width linescan is placed horizontally and vertically onto every detected local maximum. The measured intensity profiles are fitted to a Gaussian function (shown in E; orange numbers #1 - #4 refer to the equally marked spots in (B)). The best Gaussian fit is selected and its full width at half maximum (FWHM) taken as maximum size. (C) A 100 nm (5 pixel) diameter ROI is placed onto the maxima location (pixel coordinates). The intensity of each maximum is measured and background corrected by the average intensity measured next to the membrane sheet (not shown). For the inter-channel intensity correlation, the same ROI is used for measuring the intensity in the other channel (and this value is background corrected by the background taken in the other channel). In addition, the fluorescence mass centers are determined in subpixel coordinates, providing a more precise position, as it circumvents pixel quantization. Maxima with a fit quality of R^2^ > 0.8 and the Gaussian peaks in the central 10 pixels of the 31-pixel long intensity profile (determined in B) are rated as clusters. The cluster positions in subpixel coordinates are used to measure distances between clusters. (D) The 100 nm ROI is enlarged to a 900 nm diameter ROI, placed onto the maxima positions as before the 100 nm ROI. This time, we count the number of maxima in the ROI. Subtracting the value of 1 from the counted maxima yields the number of neighbored maxima of the maximum onto which the ROI was placed. (E) Intensity profiles. Orange open circles indicate data points (the average of three pixels) measured by the 31-pixel long and 3-pixel width linescan. Blue lines indicate the fitted Gaussians.


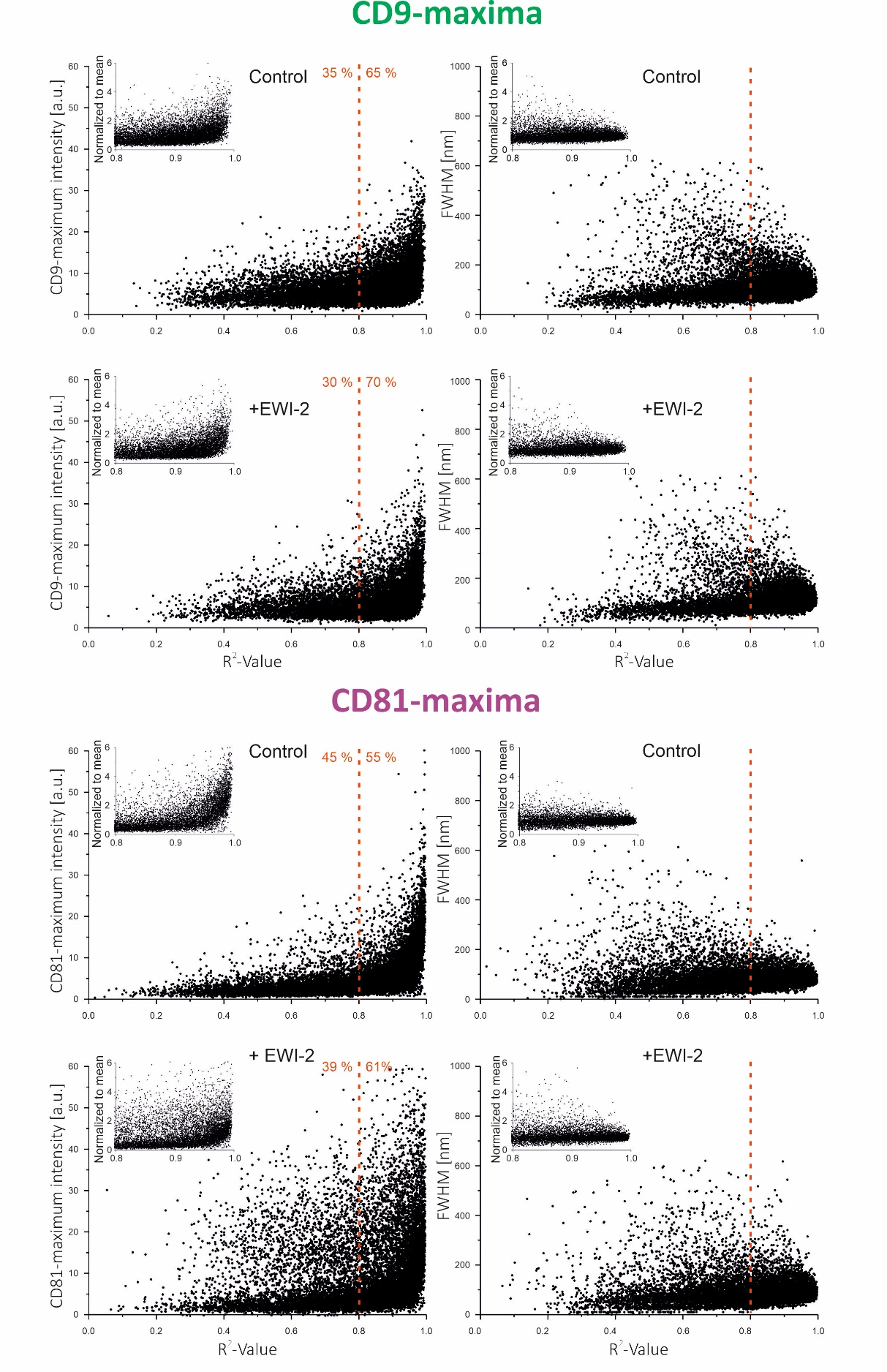


**Supplementary Figure 3.** *Correlation between CD9-/CD81-maxima intensity/FWHM and R^2^.* Maxima of Figure 1F and G, plotting maxima intensity or FWHM versus the R^2^ value of the Gaussian fit to the maxima intensity distribution (for example fits, see Supplementary Figure 2E). For clarity, no intensity values above 60 a.u. are shown. Insets show values normalized to the mean. In the population of maxima with an R^2^ > 0.8, maxima intensities but not FWHM tend to increase with the R^2^ value.

Upper panels, CD9 maxima. ‘Control’, 15,460 maxima (7.94 maxima/µm^2^); ‘+ EWI-2’, 11,433 maxima (6.39 maxima/µm^2^). The percentage of maxima with an R^2^ > 0.8 is 65 % (‘Control’) and 70 % (‘+ EWI-2’), from these, most have a centered Gaussian peak and are rated as clusters (see text for the criteria of rating maxima as clusters; 64 % (‘Control’) and 68 % (‘+ EWI-2’)). EWI-2 elevation increases the average CD9-maxima intensity by 1.6 %. Maxima with an R^2^ > 0.8 are 37 % (‘Control’) and 38 % (‘+ EWI-2’) brighter than maxima with an R^2^ ≤ 0.8.

Lower panels, CD81-maxima. ‘Control’, 12,800 maxima (6.54 maxima/µm^2^); ‘+ EWI-2’, 13,021 maxima (6.38 maxima/µm^2^). The percentage of maxima with an R^2^ > 0.8 is 55 % (‘Control’) and 61 % (‘+ EWI-2’), from these most fulfill the criteria of a centered peak and are rated as clusters (53 % (‘Control’) and 57 % (‘+ EWI-2’)). EWI-2 elevation increases the average CD81-maxima intensity by 79.4 %. Maxima with an R^2^ > 0.8 are 174 % (‘Control’) and 59 % (‘+ EWI-2’) brighter than maxima with an R^2^ ≤ 0.8.


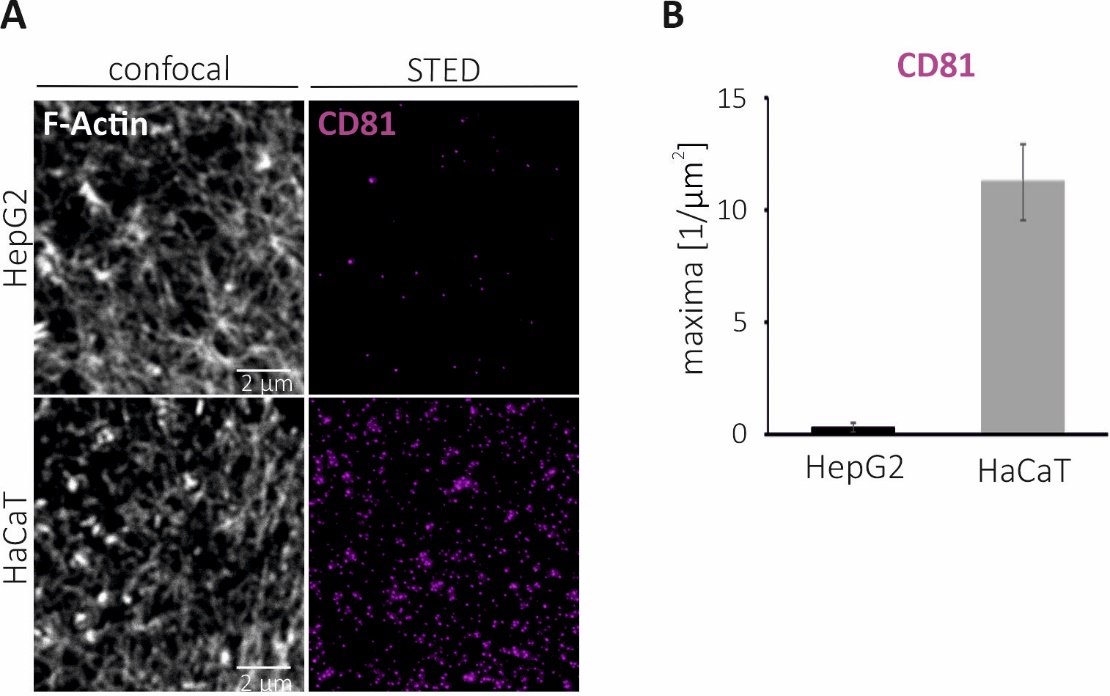


**Supplementary Figure 4.** *CD81-maxima density in CD81-lacking HepG2 and CD81-expressing HaCaT cells.* (A) Membrane sheets generated from HepG2 (top) and HaCaT cells (down). Membranes are fixed and stained for CD81. During image acquisition, membranes were identified via F-actin staining (with Phalloidin-iFluor 488). Shown are confocal micrographs of the F-actin (Phalloidin-iFluor 488, shown in grayscale) and STED micrographs of CD81 (STAR RED; shown in magenta). (B) CD81-maxima density. In HaCat T cells, the antibody recognizes 40-fold more maxima than in HepG2 cells that do not express CD81. Values are given as means ± SD (n = 2 and 3 biological replicates for HepG2 and HaCaT cells, respectively).


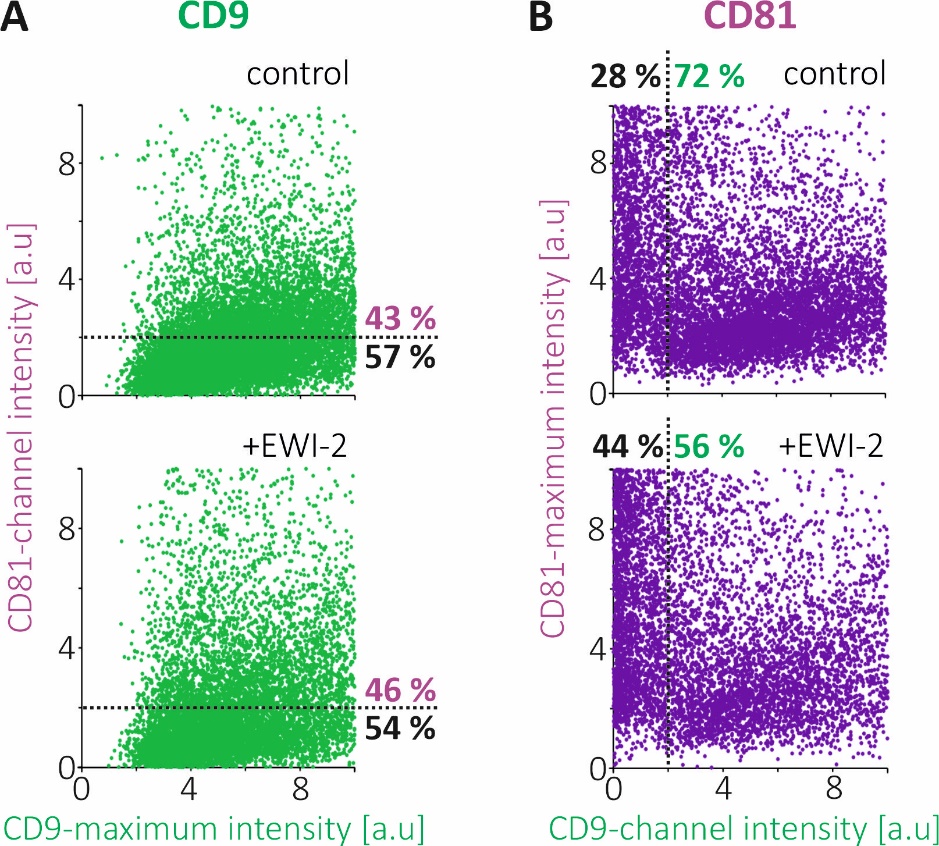


**Supplementary Figure 5.** *Magnified views from Figure 1F and G.*


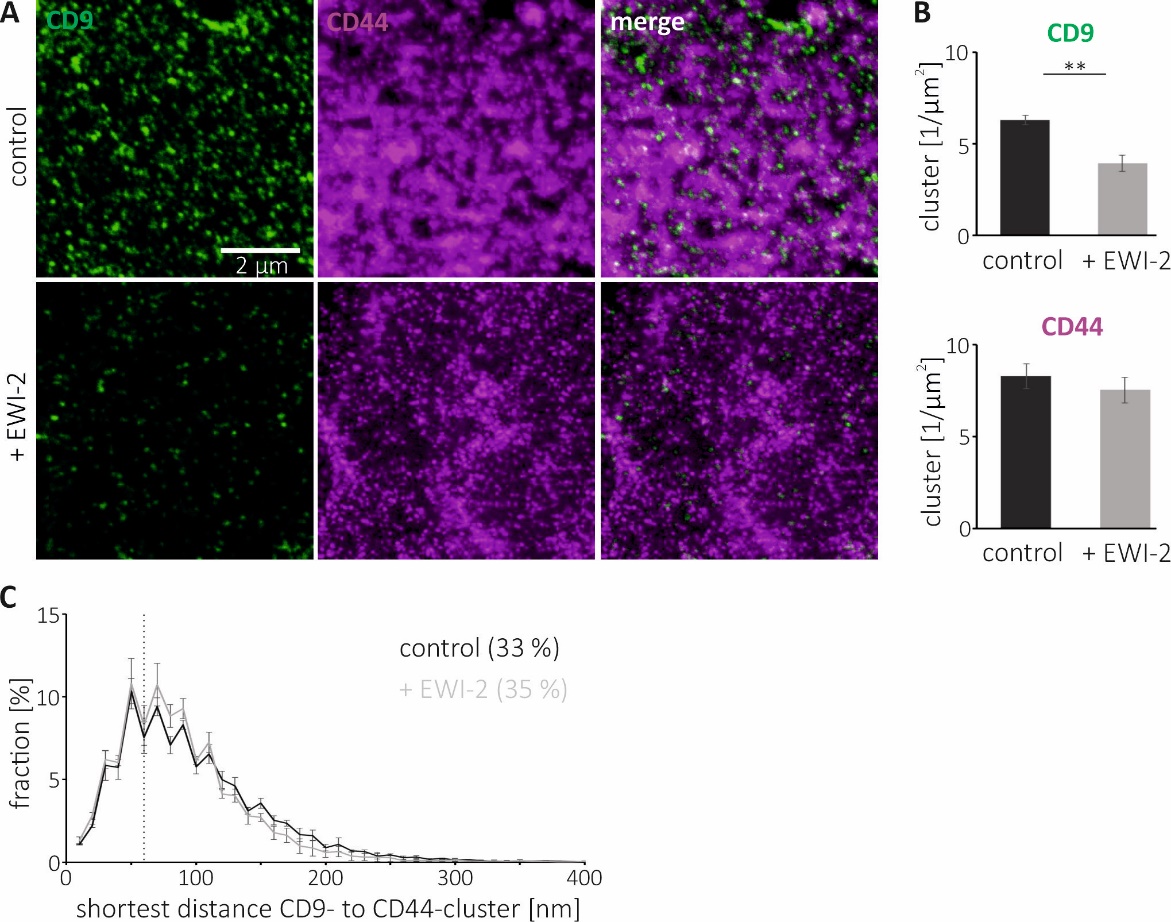


**Supplementary Figure 6.** *Cluster density and shortest inter-cluster distance of CD9 and CD44 in control cells and after EWI-2 elevation.* (A) As in Figure 1, but analysis of CD9 (Alexa594; shown in green) and CD44 (STAR RED; shown in magenta). Shown are STED micrographs of membrane sheets from HaCaT cells (‘control’) and EWI-2-GFP overexpressing cells (‘+ EWI-2’). Shown are anecdotal images, as the CD44 staining-pattern is highly variable between membrane sheets. Sometimes, maxima are well-defined and spaced, similar as in CD9 images (not shown); sometimes, well-defined maxima densely populate certain areas, but are excluded from others (see lower CD44 panel); sometimes, maxima are rather fuzzy and locate in areas that have an apparently uniform fluorescence background (see upper CD44 panel). Compared to other analysed proteins, CD44 differs, as the density of maxima exceeds the density of clusters (only about a third of the maxima are rated as clusters). (B) CD9- and CD44-clusters density. (C) Percentage of CD9-clusters plotted against distance to the next nearest CD44-clusters.


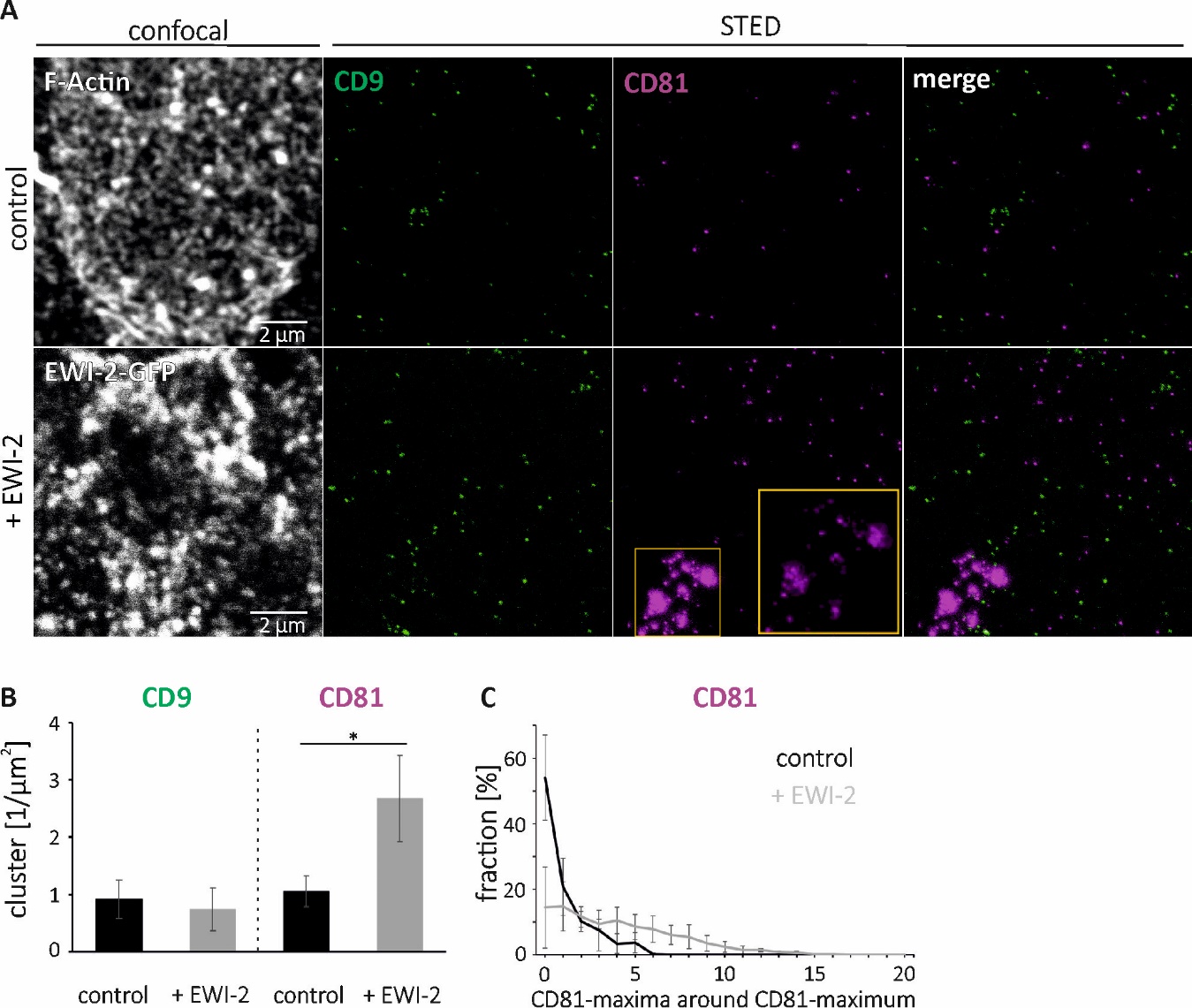


**Supplementary Figure 7.** *Cluster density and number of neighbored-maxima in Jurkat T cells.* (A) Confocal and STED micrographs of membrane sheets generated from Jurkat T cells (‘control’) or EWI-2-GFP overexpressing Jurkat T cells (‘+ EWI-2’). Membranes are fixed and stained for CD9 (Alexa594; shown in green) and CD81 (STAR RED; shown in magenta). During image acquisition, membranes from control and EWI-2 overexpressing cells were identified via F-actin staining (with Phalloidin-iFluor 488) and GFP, respectively (shown in gray). (B) CD9- and CD81-clusters density. (C) Percentage of CD81-maxima plotted against the number of their neighbored-maxima located in a 900 nm diameter circle. Values are given as means ± SD (n = 3 biological replicates, 10 – 17 membrane sheets per biological replicate).


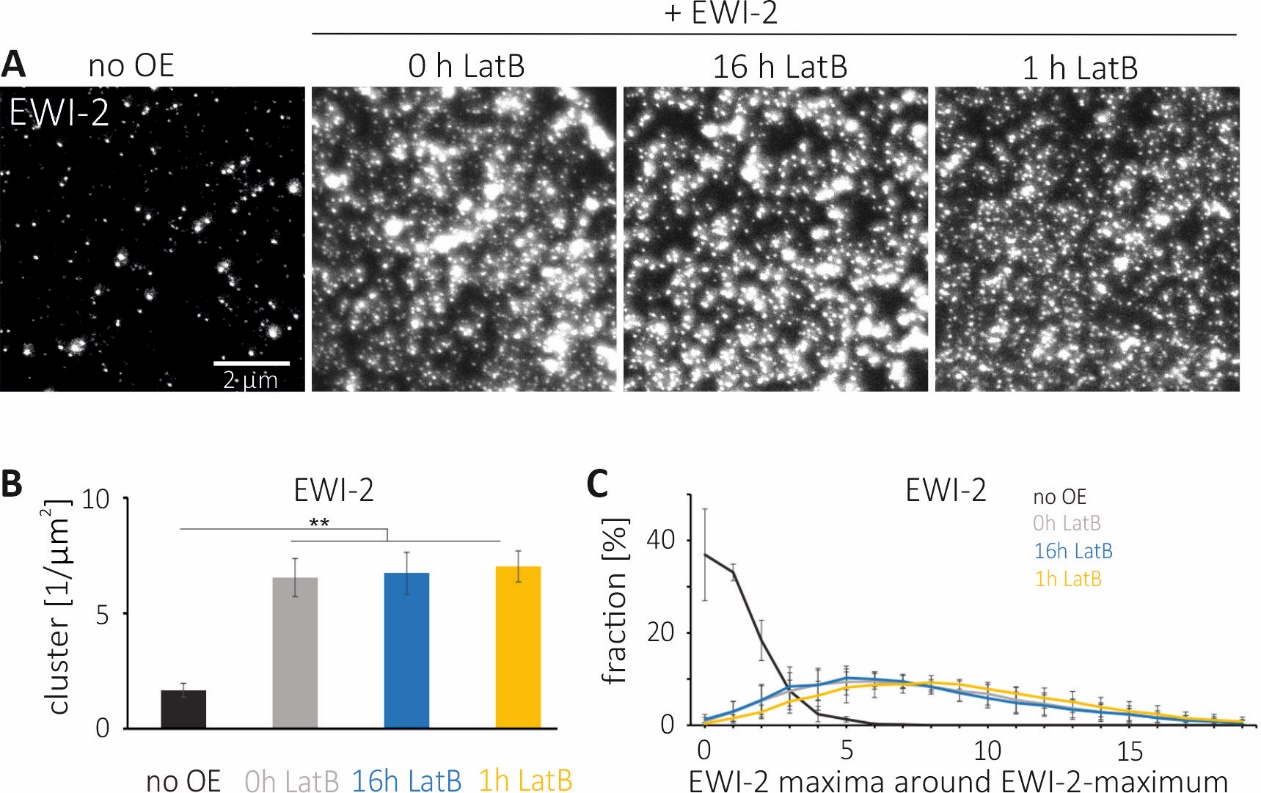


**Supplementary Figure 8.** *Cluster density and neighbored-maxima of EWI-2 after Latrunculin B treatment.* (A) Membrane sheets from HaCaT cells not overexpressing (no OE) and overexpressing EWI-2-GFP (+ EWI-2; 0h, 16h, 1h LatB). Overexpressing cells were not incubated (0 h) or incubated with 25 µM Latrunculin B for 16 h or 1 h prior to membrane sheet generation. Membrane sheets were fixed and double stained for EWI-2 and CD81. (not shown). (B) EWI-2-clusters density. (C) Percentage of EWI-2-maxima plotted against the number of their neighbored-maxima within a 900 nm diameter circle. Overexpression of EWI-2 more than doubles the EWI-2-cluster density and therefore shifts the peaks of the neighbored-maxima distributions towards larger values. Values are given as means ± SD (n = 3 biological replicates).
